# Hydrogen Bonding is Required for Continuous Polyvinylpyrrolidone Adhesive Films

**DOI:** 10.1021/acs.langmuir.6c04951

**Published:** 2026-10-08

**Authors:** Mahira Mim, An C. N. Nguyen, Mamie Mulder, Hanae Takahashi, Mackenzie Windus, Wei Chen

**Affiliations:** Chemistry Department, Carr Laboratory, 7397 Mount Holyoke College, 50 College Street, South Hadley, Massachusetts 01075, United States

## Abstract

The versatility of polyvinylpyrrolidone (PVP) as a dispersing and adhesive agent lies in its amphiphilicity and hydrogen bonding capability. In this study, silicon wafers (SiO_2_) were silanized with *N*-(2-aminoethyl)-3-aminopropyltriethoxysilane (AEAPTES), (*N*,*N*-dimethylamino)­trimethylsilane (TMS), and n-octyldimethylchlorosilane (C8) to construct four substrates with varying fractions of hydrogen bonding sites and hydrophobic groups. PVP thin films were fabricated on the substrates using both static adsorption and spin coating methods. PVP spontaneously adsorbs to and results in 1 nm-thick films on all the substrates, demonstrating the polymer’s capability as a universal adhesive. The continuity of the dried PVP films at the nanoscopic and micron scales was imaged using atomic force and optical microscopy, respectively. The film stability was correlated to the fraction of substrate hydrogen bonding sites. The films are stable on SiO_2_ with 100% hydrogen bonding sites, unstable on TMS with 100% hydrophobic groups, and metastable on AEAPTES and C8 with a mixture of hydrogen bonding sites and hydrophobic groups.

## Graphical abstract

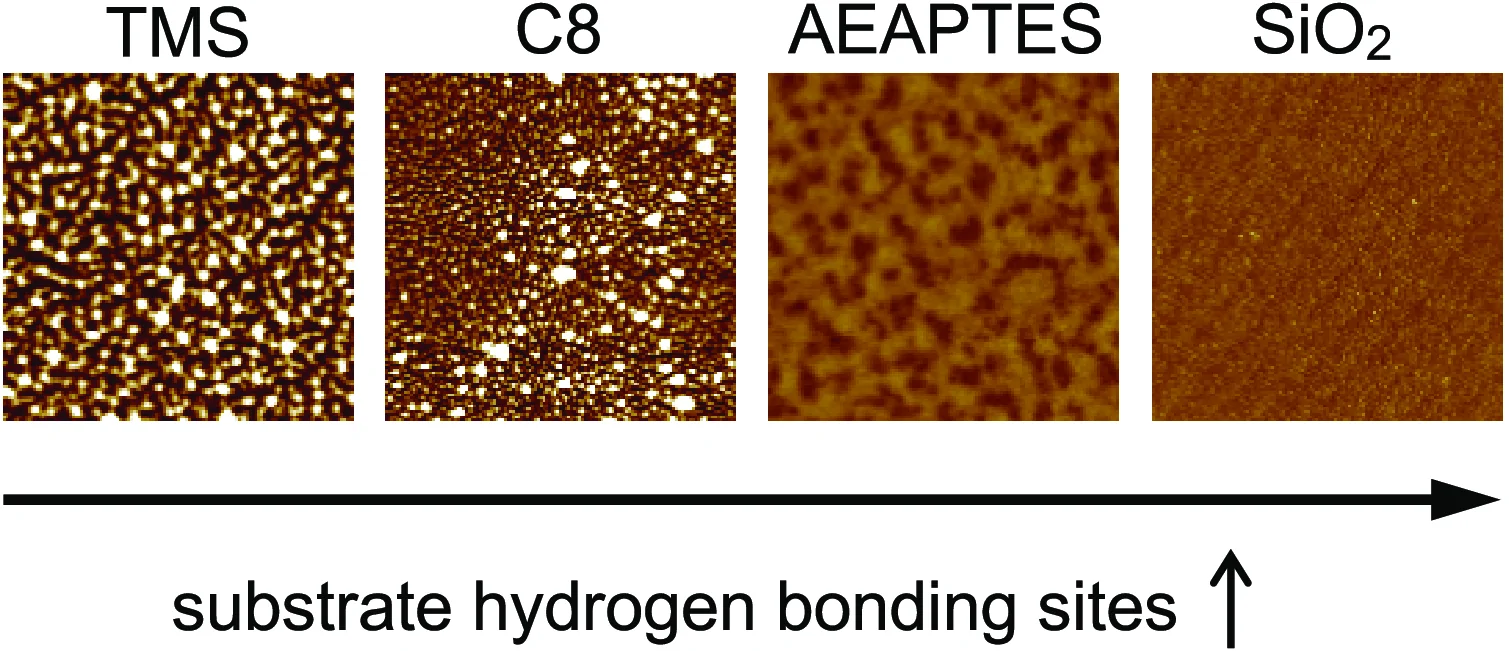

## Introduction

Polyvinylpyrrolidone (PVP) is widely used in pharmaceutical, cosmetic, medical, sensing, energy, and construction sectors due to its low cost, nontoxicity, solubility in water and polar organic solvents, hydrophilicity, and biocompatibility.^1–4^ In particular, PVP has excellent dispersive and adhesive properties attributed to its amide and hydrocarbon moieties that are capable of hydrogen bonding and hydrophobic interactions, respectively.^5–8^

PVP adsorption onto silica particles has been extensively investigated, most notably as a function of solution pH^5,8,9^ and PVP molecular weight.^5,10–12^ At low pH, the adsorption is driven by hydrogen bonding between surface silanol groups and carbonyl groups in PVP. As pH increases into the alkaline region, silanol groups become deprotonated and PVP adsorption is suppressed.^5,8^ Yu et al. reported that the adsorbed amount on silica particles had a positive correlation with PVP molecular weight.^12^ Low molecular weight polymers are confined in a flat conformation (mostly trains), whereas higher molecular weight polymers adopt more extended conformations (more loops and tails).^12,13^ The adsorbed amount of 40 kDa PVP on silica was ∼1 mg/m^2^,^12^ corresponding to ∼1 nm of film thickness. In conjunction with the previously reported PVP chain width of 1 nm,^9^ adsorbed polymers mostly lie flat, maximizing hydrogen bonding interactions with the silanol groups on silica. Beyond silica, PVP also spontaneously adheres to other colloidal particles.^14–20^ The driving forces include hydrogen bonding, hydrophobic interactions, and electrostatic attractions, depending on substrate’s chemical functionalities.^21^ It is noteworthy that Parnas et al. studied PVP adsorption on silica particles modified with various alkyl silanes and observed that the adsorbed amount decreased slightly in order of vinyl > ethyl > isobutyl, untreated.^14^ The difference was attributed to different interactions between the carbonyl groups in PVP and the various surface functional groups.^14^ PVP adsorption has also been carried out on flat substrates, such as silicon wafers,^8,9,12^ to allow for the comparison of different cleaning methods and certain quantification techniques, including optical reflectometry and quartz crystal microbalance. Although there are aforementioned reports on adsorbed amounts on various substrates and adsorbed chain conformations, there is only one report on PVP film morphology using imaging techniques.^22^ In the study, nonequilibrium morphologies of spin-coated PVP films on polydimethylsiloxane substrates under various conditions were probed using optical and atomic force microscopy.^22^ We are not aware of any prior work on nanoscopic and microscopic morphologies of spontaneously adsorbed PVP films and correlating PVP morphology with substrate chemical composition.

Film morphology and stability have paramount implications in material properties and performance. The thermodynamic stability of thin films has been comprehensively examined.^23–35^ The effective interface potential, ϕ­(*h*), represents the excess free energy per unit area that is necessary to reduce the separation between solid–liquid and liquid–gas interfaces from infinity to a finite film thickness, *h*.^32^ When ϕ­(*h*) remains positive as a function of thickness, a film–substrate system is stable. This signifies that the films are continuous and pinhole-free in the entire thickness range.^32^ Conversely, when ϕ­(*h*) has a global minimum, the corresponding system becomes unstable, and the films dewet (rupture) to form discontinuous morphologies.^32^ A metastable system exhibits instability at low film thicknesses, but attains stability at higher film thicknesses, undergoing a discontinuous to continuous morphological transition with increasing thickness.^32^ Film continuity as a function of thickness thus provides a framework to distinguish between stable, unstable, and metastable systems.

Spin coating is a versatile technique to fabricate polymer thin films on flat substrates. The Meyerhofer equation, *h* = kω^–1/2^, allows the mathematical prediction of film thickness (*h*) as a function of spin rate (ω) and initial solution viscosity (k) in the wetting regime.^36^ In our recent work, we demonstrated failure of the model when spontaneous polymer adsorption at the substrate–solution interface is significant and when polymer thin films dewet during solvent evaporation.^37^ Instead of the Meyerhofer exponent of −1/2, experimentally obtained exponents deviated from −1.3 to 0. Notably, the exponent of 0 was observed when the polymer–substrate system was unstable.^37^ A modified Meyerhofer model that decouples the total spin-coated thickness into two components – the spontaneously adsorbed thickness (*h*_1_) and the spin-deposited thickness (*h*_2_) – was proposed. The former largely depends on polymer–substrate interactions, while the latter is mostly dependent on polymer concentration and spin rate. Accordingly, static adsorption and adsorptive spin coating methods were implemented to quantify *h*_1_ and *h* (*h*_1_ + *h*_2_) values, respectively.^37^

In this work, silicon wafers (SiO_2_) were modified with three functional silanes: (*N*,*N*-dimethylamino)­trimethylsilane (TMS), *N*-(2-aminoethyl)-3-aminopropyltriethoxysilane (AEAPTES), and n-octyldimethylchlorosilane (C8). PVP thin films were fabricated on these substrates via static adsorption and adsorptive spin coating. Static adsorption yielded 1 nm-thick PVP films, independent of polymer–substrate interactions. However, morphologies and stabilities of spin-coated PVP films were strongly influenced by film thickness and PVP-substrate interactions. For the first time, the crucial role of hydrogen bonding in producing metastable and stable PVP films is revealed by systematically controlling substrate chemistry and judiciously utilizing imaging techniques.

## Experimental Section

### Materials

Silicon wafer substrates were obtained from International Wafer Service (100 orientation, P/B doped, 1–10 Ω cm resistivity, 475–575 μm thickness). Silanes, including (*N*,*N*-dimethylamino)­trimethylsilane (TMS-NMe_2_), *N*-(2-aminoethyl)-3-aminopropyltriethoxysilane (AEAPTES), and n-octyldimethylchlorosilane (C8), were purchased from Gelest. Polyvinylpyrrolidone (PVP, 40 kDa) was acquired from Sigma-Aldrich. ACS-grade toluene and ethanol were from Fisher Scientific. Oxygen (99.999%) and nitrogen (99.998%) gases were supplied by Airgas and Ivey Industries, respectively. Ultrapure water (resistivity ≥ 18.2 MΩ·cm) was purified using a Millipore Milli-Q Biocel System from Millipore Corp. Glassware was cleaned in a base bath consisting of potassium hydroxide dissolved in a mixture of water and isopropyl alcohol, rinsed thoroughly with deionized water, and stored in a clean oven at 110 °C before use. All samples were dried and stored in a CaSO_4_ desiccator until further use.

### Instrumentation and Characterization

Size and dispersity of PVP chains in solution were assessed by dynamic light scattering (DLS, Malvern Zetasizer Nano-S, 4 mW He–Ne laser, λ = 632.8 nm). Refractive indices of PVP (*n* = 1.520) and water (*n* = 1.330), and viscosity of water (η = 0.8872 cP) at 25 °C were used. Silicon wafers and glassware were dried in a Precision 51221126 Gravity Convection Lab Oven (Thermo Fisher Scientific). Silicon wafers were treated with oxygen plasma using a PDC-001 Harrick plasma cleaner. Spin coating was performed on a Laurell WS-650MZ-23NPPB single-wafer spin processor. Thickness measurements were carried out by ellipsometry (Gaertner Scientific LSE Stokes ellipsometer with a λ = 632.8 nm He–Ne laser and a 70° incident angle between the light source and the surface normal), using the following refractive indices: *n*_0_ = 1 for air, *n*_1_ = 1.46 for silicon oxide, silane and polymer layers, and *n*_s_ = 3.85 and *k*_s_ = −0.02 for silicon. Heat treatment was performed in a J-KEM Scientific heater. Dynamic advancing (θ_ad_) and receding (θ_re_) water contact angle measurements were performed on a Ramé-Hart telescopic goniometer with a Gilmont syringe and a 24-gauge flat-tipped needle. Nanoscopic topography was imaged by tapping-mode atomic force microscope (AFM, Veeco Metrology Dimension 3100, silicon tip). The wave-like patterns in the background of some images are due to optical interference between the AFM laser and reflective sample surfaces. Micron-scale surface morphology was captured in reflective darkfield mode on an Olympus BX51 optical microscope. Some hue variations in optical images are artifacts caused by differences in instrument settings. For both imaging techniques, multiple images from several samples of each type were acquired, and representative images were chosen.

### PVP Solutions

0.1 and 0.2 wt % PVP solutions were prepared by dissolving desired amounts of PVP powder in 50 mL of Milli-Q water. The solutions were stirred at room temperature for 24 h and had pH values of ∼6. After equilibrating for a few days, they were analyzed using DLS to confirm complete polymer dissolution. The hydrodynamic diameter and polydispersity index were 17 ± 1 nm and 0.28 ± 0.07, respectively.

### Substrate Preparations

Silicon wafers were cut into 1.4 × 1.4 cm^2^ squares, rinsed with deionized water, and dried with compressed air. Substrates were then dried in the oven at 110 °C for 30 min, treated with oxygen plasma for 15 min (∼300 mTorr, 30 W), and allowed to equilibrate in the reactor for 15 min. Cleaned substrates were immediately used for silanization or PVP deposition. To fabricate silanized substrates, clean silicon wafers were placed in a custom holder inside a Schlenk tube containing ∼0.5 mL of a silane, ensuring no direct contact between the liquid and the substrates. The sealed tube was heated in an air bath at 70 °C for 24 h or 15 min. After the reaction, samples in the holder were submerged in toluene. They were sequentially rinsed with toluene (4×), ethanol (3×), and Milli-Q water (3×) to remove unreacted silanes and other physiosorbed species, followed by drying thoroughly with nitrogen gas. The AEAPTES-modified substrates were additionally treated at 110 °C for 15 min.

### Static Adsorption and Adsorptive Spin Coating of PVP

A desired amount of PVP solution (100 μL on SiO_2_ and 400 μL on silane-modified substrates) was dispensed onto a substrate and allowed to adsorb for 1 min. For static adsorption, samples were triple rinsed with 1 mL of Milli-Q water using a micropipette and dried under a nitrogen stream. Alternatively, spin-coated PVP layers were fabricated by spinning the samples at a desired rate (900 to 6000 rpm) for 1 min under nitrogen.

### Thermal Annealing of PVP

Individual PVP samples were placed in scintillation vials. In some vials, 20 μL of Milli-Q water was added without direct contact with the sample. The vials were capped and heated at 150 °C for 15 min.

## Results and Discussions

### Substrates

Silicon wafers consist of an oxide layer (SiO_2_) after oxygen plasma treatment. In addition, three silanized substrates with distinct surface chemical compositions were prepared by reacting silicon wafers with (*N*,*N*-dimethylamino)­trimethylsilane (TMS), *N*-(2-aminoethyl)-3-aminopropyltriethoxysilane (AEAPTES), and n-octyldimethylchlorosilane (C8) at 70 °C for 24 h. Chemical structures of polyvinylpyrrolidone and four substrates are shown in Figure 1.

**Figure 1:**
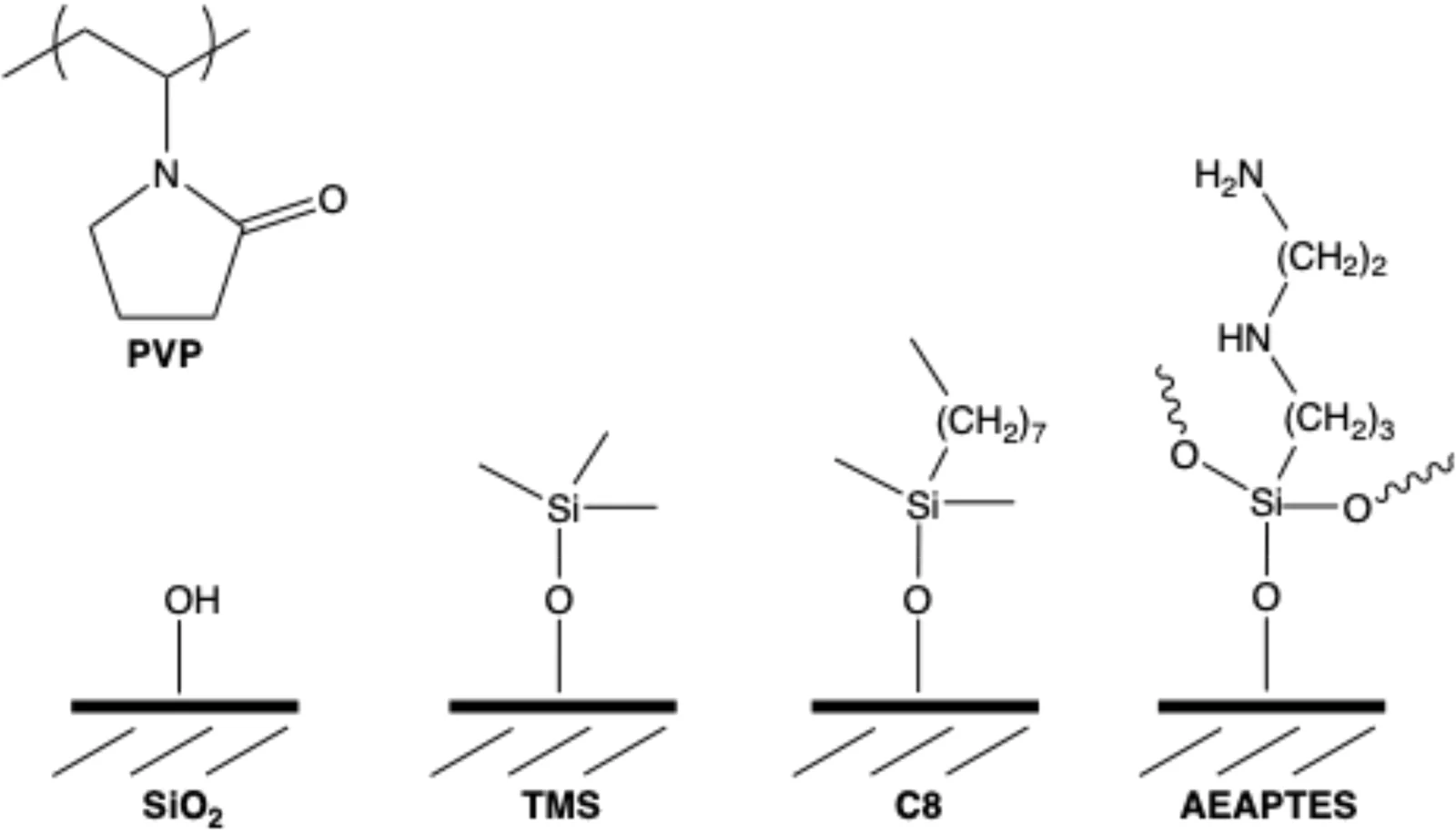
Chemical structures of polyvinylpyrrolidone (PVP) and four substrates. Silicon wafer (SiO_2_) and silicon wafers modified with (*N*,*N*-dimethylamino)­trimethylsilane (TMS), *N*-(2-aminoethyl)-3-aminopropyltriethoxysilane (AEAPTES), and n-octyldimethylchlorosilane (C8).

The four substrates were fully characterized, as shown in Table 1. The thickness values of TMS and C8 indicate that they are monolayers in nature, whereas the significantly thicker AEAPTES layer is attributed to the vertical as well as lateral attachments of silane molecules through the reactive triethoxy functional groups.^38^ The AFM images of the substrates are smooth and featureless (Table 1S). Advancing (θ_ad_) and receding (θ_re_) water contact angles indicate that SiO_2_ is extremely hydrophilic with contact angles of 0°/0° (θ_ad_/θ_re_) and TMS is the most hydrophobic with contact angles of 104°/89°. C8 is slightly less hydrophobic than TMS, most likely due to the steric hindrance of the large octyl groups, which limits access to the surface silanol groups and results in a reaction yield of less than 100%. The presence of hydrophilic amine groups and hydrophobic methylene groups gives rise to AEAPTES’s moderate hydrophilicity.

**Table 1:** Thickness (T), Advancing, Receding, and Average Water Contact Angles (θ_ad_, θ_re_, θ_avg_), and Surface Coverage of Hydrophobic Moieties (f_1_) of Different Substrates

	SiO_2_	TMS	C8	AEAPTES
T (nm)	0.9 ± 0.1	0.5 ± 0.1	0.6 ± 0.1	4.4 ± 0.3
θ_ad_ (°)	0 ± 2	104 ± 2	100 ± 3	46 ± 3
θ_re_ (°)	0 ± 2	89 ± 2	79 ± 4	11 ± 2
θ_avg_ (°)	0 ± 2	96.5 ± 2	89.5 ± 3	29 ± 2
f_1_	∼0	∼1	∼0.93	∼0.15

Surface coverage of C8 was determined using the Israelachvili eq (eq 1)^39^ and treating the substrate as a binary composite consisting of f_1_ surface fraction of hydrophobic component 1 (−CH_3_) and f_2_ surface fraction of hydrophilic component 2 (−OH), with f_1_ + f_2_ = 1. θ, θ_1_, and θ_2_ are respective contact angles of the composite, component 1, and component 2. θ_2_ = 0° based on contact angles of silicon wafers. In a recent study, we showed that average contact angles (θ_avg_) provided more meaningful surface composition values than advancing or receding contact angles.^37^ The reaction between TMS and silicon wafers is expected to be fast and quantitative, converting all −OH groups to −CH_3_ groups. Thus, the average contact angle of TMS was used as θ_1_ = 96.5°. Surface coverage of C8 was calculated to be 0.93, indicating that 7% of the initial −OH groups remain unreacted. Similar calculations were performed on AEAPTES, assuming that the amine groups have a 0° contact angle and that the −CH_2_– group has a similar contact angle to the −CH_3_ group. The last row in Table 1 indicates that the fractions of the hydrophobic groups are 0% on SiO_2_, 100% on TMS, 93% on C8, and 15% on AEAPTES, yielding corresponding hydrogen bonding site fractions of 100%, 0%, 7%, and 85%, respectively.

1 $${\left(1+\cos\theta \right)}^{2}=f_{1}\;{\left(1+{\cos\theta }_{1}\right)}^{2}+f_{2}\;{\left(1+{\cos\theta }_{2}\right)}^{2}$$

### Static Adsorption

Static adsorption was utilized to extract the spontaneously adsorbed thickness, *h*_1_, on the four substrates. It was carried out using a 0.1 wt % PVP solution at room temperature for 1 min. Intriguingly, the spontaneously adsorbed layers have an identical value of 1.0 ± 0.1 nm on all the substrates, as shown in Table 2. Longer adsorption times and higher PVP concentrations did not yield thicker PVP films (not shown here). The 1 nm thickness value is in excellent agreement with the PVP chain width reported in the literature,^9^ suggesting that statically adsorbed PVP chains adopt a flat conformation with very few loops and tails. We can infer that whether PVP adsorption is driven by 100% hydrogen bonding on SiO_2_, or 100% hydrophobic interactions on TMS, or a mixture of the two on AEAPTES and C8, the adsorbed amount and the chain conformation are indistinguishable on the four substrates examined in this study. This result is a confirmation that PVP spontaneously adheres to a diverse range of substrates via its hydrogen bonding and/or hydrophobic components.

**Table 2:**
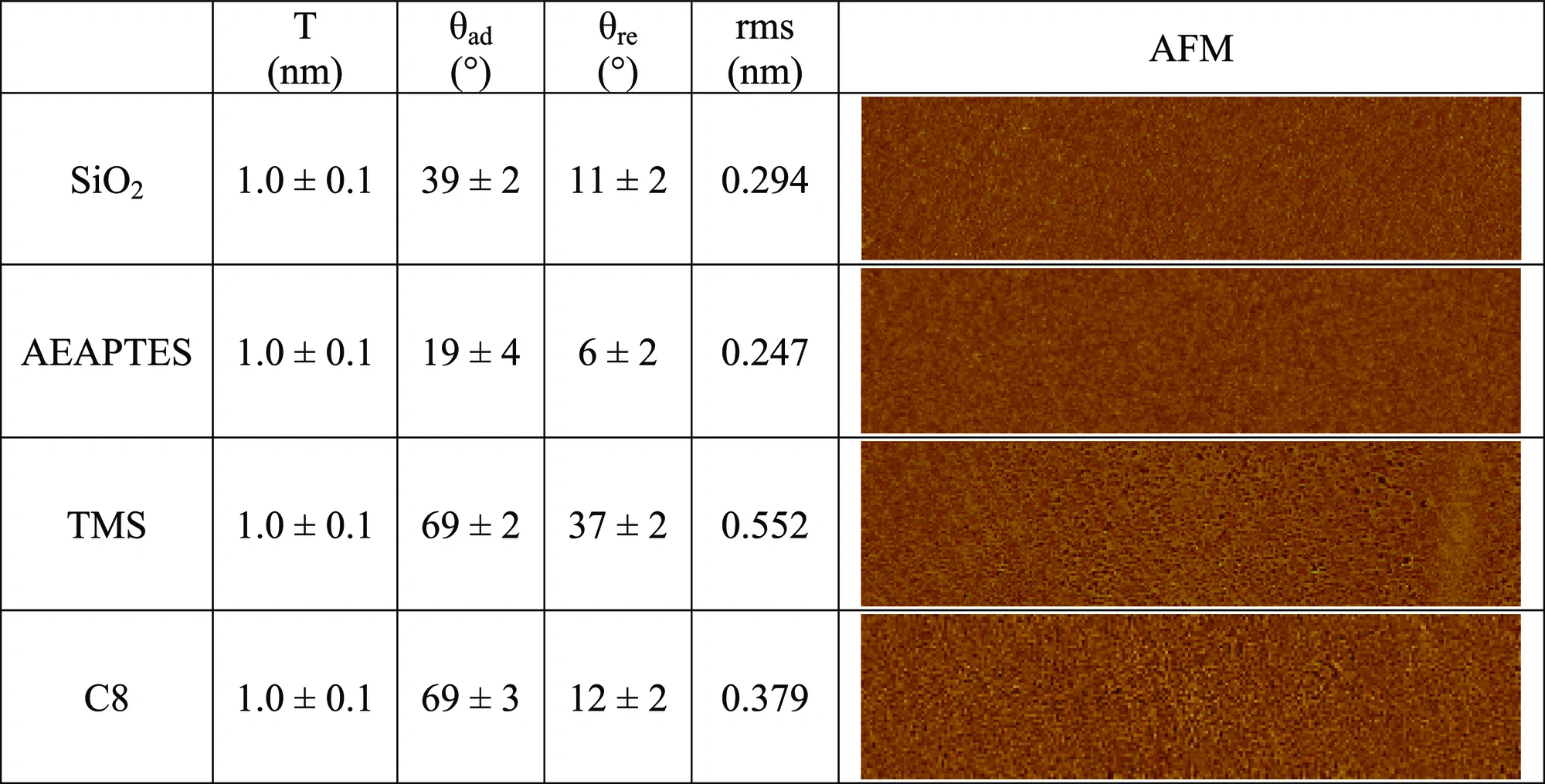
Thickness (T), Advancing and Receding Water Contact Angles (θ_ad_, θ_re_), AFM Root-Mean-Square Roughness (rms), and AFM Images (Size: 5 × 1.25 μm^2^; Data Scale: 10 nm) of PVP Films After 1 Min Static Adsorption Using 0.1 wt % PVP Solution on Different Substrates

While AFM images of the statically adsorbed PVP films are continuous on SiO_2_ and AEAPTES, those on TMS and C8 exhibit small holes, indicating film dewetting. Higher advancing contact angles of statically adsorbed PVP films on the two latter substrates reflect exposure of the underlying hydrophobic substrates. The differences in film continuity correlate strongly with substrate hydrophobicity – PVP films spread uniformly on the hydrophilic SiO_2_ and AEAPTES, but break up on the hydrophobic TMS and C8. The indistinguishable thicknesses and substrate-dependent differences in continuity of spontaneously adsorbed PVP films prompted further investigation of PVP film stability on the different substrates.

### Spin Coating

Metastable films exhibit dewetting at low film thicknesses and wetting at high thicknesses, which can be used to distinguish them from unstable and stable films. Spin coating provides the means to fabricate PVP films with a wide range of thicknesses by varying spin rate and polymer concentration. PVP morphology as a function of thickness allows the identification of film stability on different substrates.

Figure 2 depicts spin-coated PVP film thickness as a function of spin rate using 0.1 and 0.2 wt % PVP solutions. The spin curves on SiO_2_, AEAPTES, and C8 are superimposed in each plot. They exhibit the expected power law relationship between film thickness and spin rate, although the exponents (−0.32 and −0.41) deviate from the −1/2 predicted by Meyerhofer.^36^ According to our modified Meyerhofer model,^37^ total spin-coated thickness (*h*) consists of the spontaneously adsorbed thickness (*h*_1_) and the spin-deposited thickness (*h*_2_). As demonstrated in the previous section, PVP spontaneously adsorbs to all four substrates, producing an identical thickness of *h*_1_ = 1.0 nm, indicative of comparable polymer–substrate interaction strengths despite different interaction types. The superimposed spin curves further confirm identical *h*_2_ thickness values, suggesting similar PVP–PVP interactions in the spin-deposited layers. Comparing the total thicknesses from the two plots in Figure 2, the *h*_2_ layer thicknesses from 0.2 wt % solution are approximately twice of their counterparts from 0.1 wt % solution on SiO_2_, AEAPTES, and C8 substrates (not shown here). This result points to the fact that *h*_2_ is proportional to polymer concentration, consistent with our earlier report.^37^

**Figure 2:**
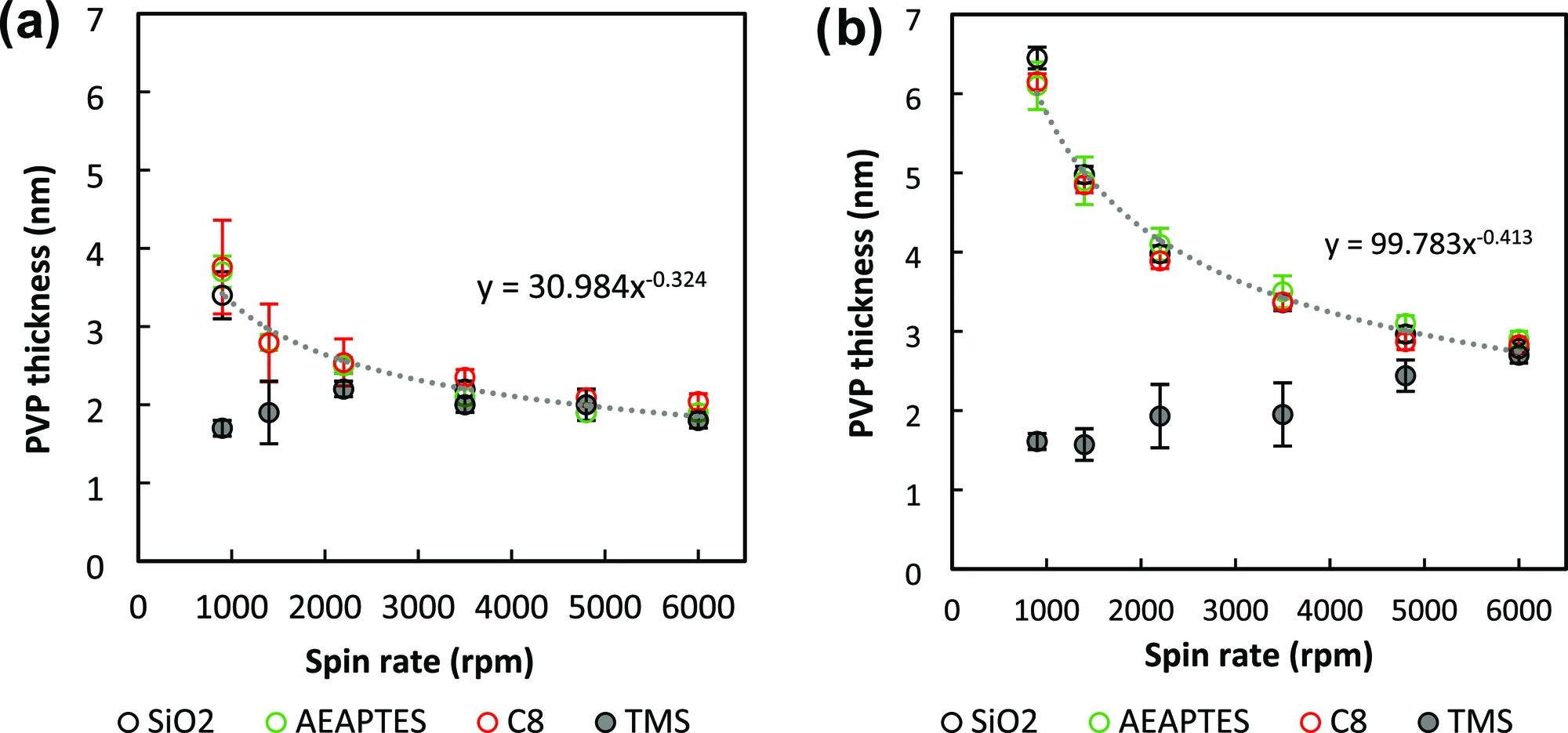
Spin-coated PVP film thickness (nm) as a function of spin rate (rpm) using (a) 0.1 wt % and (b) 0.2 wt % PVP solutions on SiO_2_, AEAPTES, C8, and TMS.

One of the most noticeable features in both plots is that the TMS spin curves demonstrate little dependence on spin rate, implicating PVP instability based on one of our earlier studies.^37^ Considering that the total PVP film thickness on TMS is ∼2 nm and the *h*_1_ layer is 1 nm, the *h*_2_ layer is ∼1 nm, independent of spin rate. The nanoscopic and microscopic morphologies of spin-coated PVP films from 0.1 wt % solution on TMS are shown in Table 3. The AFM images depict similar nanodroplet features across all spin rates, which are attributed to the dewetting of the *h*_1_ layer. The optical images consist of micron-scale dewetted features including ribbons and droplets, arising from the dewetting of the *h*_2_ layers. High advancing and receding water contact angles of ∼83°/∼42° (θ_ad_/θ_re_, Figure 1S) are verifications of the exposed hydrophobic TMS substrates. Data from 0.2 wt % solution exhibit the same trends (Table 2S, Figure 1S). The extensive dewetting on both nanoscopic and microscopic scales of spin-coated PVP films confirms that PVP on TMS is an unstable system. In an earlier work, we established that polymer film dewetting takes place during the drying process.^40^ The high interfacial free energy between the hydrophobic substrate and hydrophilic polymer can cause the aggregation of PVP chains and the exposure of underlying substrate during solvent evaporation. High rotational freedom of siloxane bonds in the TMS layer may further exacerbate the PVP film instability.

**Table 3:**
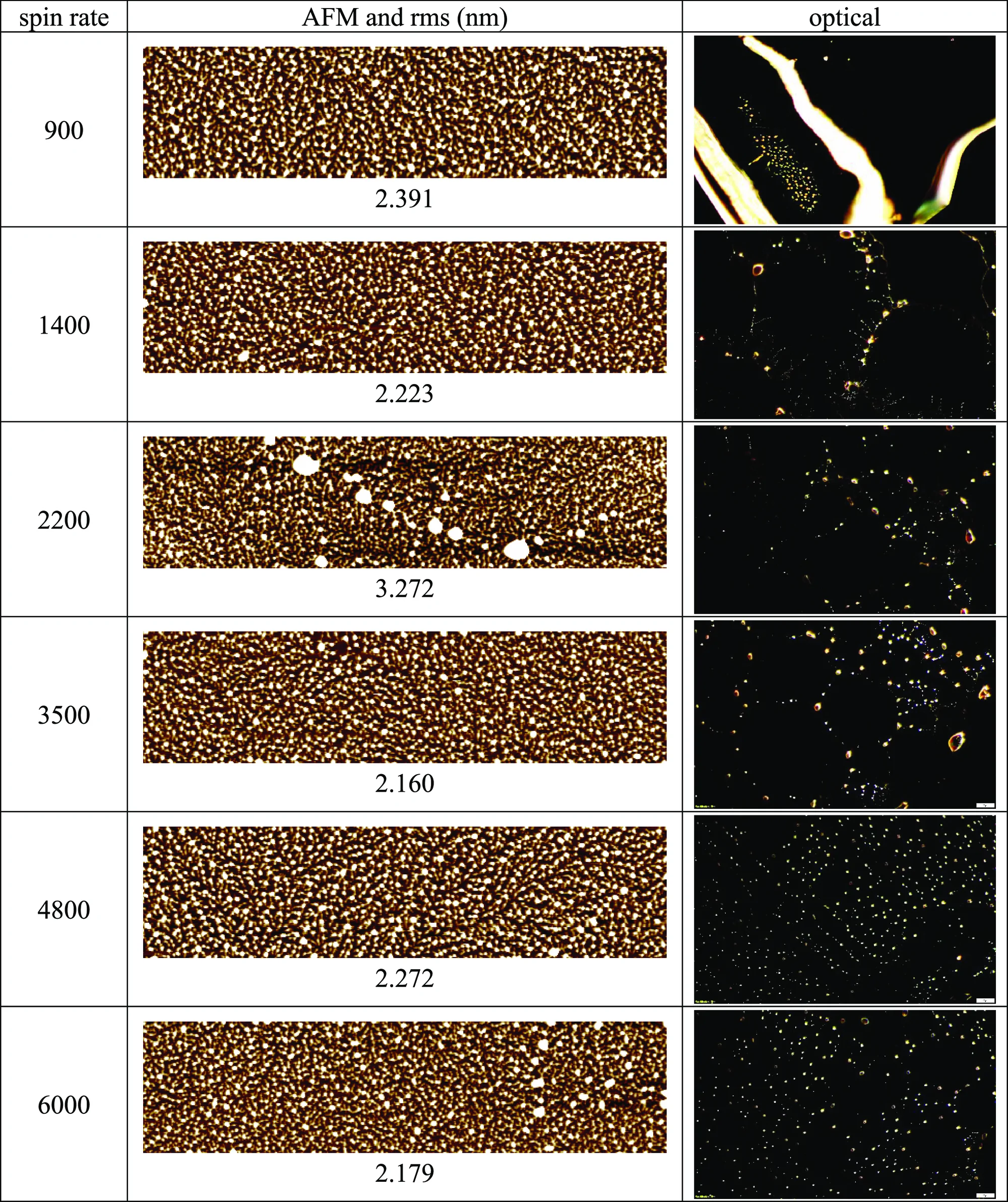
AFM Images (Size: 5 × 1.25 μm^2^; Data Scale: 10 nm), AFM Root-Mean-Square Roughness (rms), and Optical Images (Size: 362 × 226 μm^2^) of Spin-Coated PVP Films on TMS as a Function of Spin Rate (rpm) Using 0.1 wt % PVP Solution

Conversely, spin-coated PVP films completely wet the silicon wafer substrate, as shown in the AFM and optical images (Tables 4 and 3S). The few bright spots in the optical images are due to the presence of dust particles. The dynamic water contact angles of ∼11°/∼7° suggest extremely hydrophilic PVP films independent of thickness (Figure 2S). An interesting note is that spin-coated PVP films are more hydrophilic than their statically adsorbed counterparts with contact angles of 39°/11° (Table 1). This can be rationalized by the hydrogen bonding interactions between the carbonyl groups in PVP and the silanol groups on SiO_2_, which restrict statically adsorbed PVP chain conformations such that the more hydrophobic moieties are exposed to air. On the other hand, spin-deposited PVP chains in the outer layer have greater conformational freedom, allowing their carbonyl groups to hydrogen bond with the water probe fluid^21^ and exhibiting lower contact angles. Thermal annealing of these films at 150 °C, with and without water as a plasticizer, does not trigger film dewetting (Table 4S). This confirms that PVP on SiO_2_ is a stable system. Specifically, strong hydrogen bonding interactions between the statically adsorbed PVP layer and the SiO_2_ substrate produce a stable *h*_1_ base layer, with the spin-deposited *h*_2_ layer spreading evenly on top.

**Table 4:**
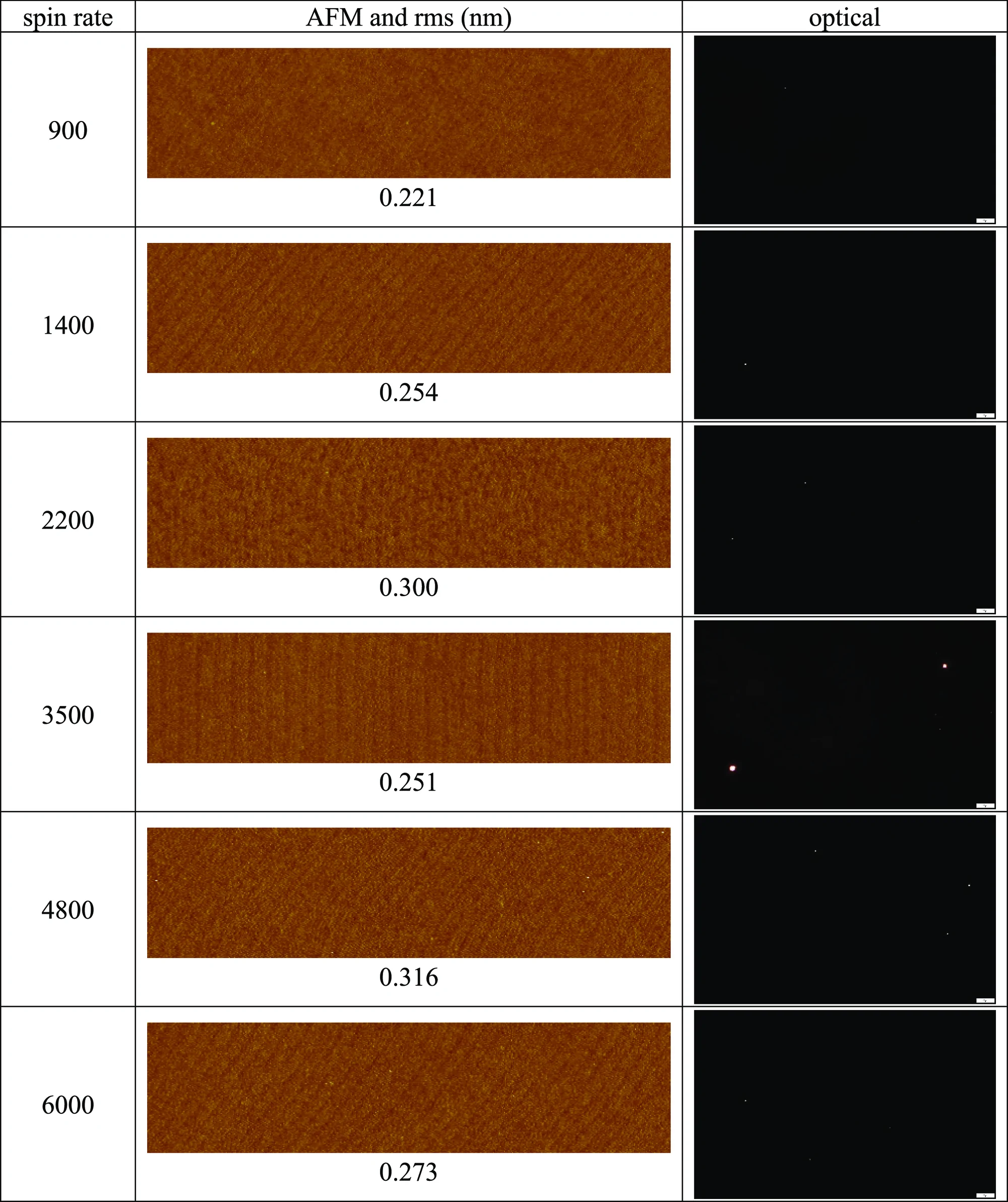
AFM Images (Size: 5 × 1.25 μm^2^; Data Scale: 10 nm), AFM Root-Mean-Square Roughness (rms), and Optical Images (Size: 362 × 226 μm^2^) of Spin-Coated PVP Films on SiO_2_ as a Function of Spin Rate (rpm) Using 0.1 wt % PVP Solution

AEAPTES substrate was chosen for its dual abilities of hydrogen bonding and hydrophobic interactions. The AFM images of spin-coated PVP films are shown in Table 5. The films from 0.1 wt % solution exhibit different extents of dewetting, with the thinner ones from high spin rates displaying small holes and the thicker ones from low spin rates revealing larger holes. Despite these morphological differences, the advancing and receding contact angles on these films are low and comparable, ∼13°/∼7° (Figure 3S). Because the underlying AEAPTES substrate is hydrophilic, exposure of the substrate upon PVP dewetting does not produce a measurable change in water contact angles. The corresponding optical images are completely featureless (Table 5S), indicating no dewetting on the microscopic scale. In contrast, the films from 0.2 wt % solution are thicker (Figure 2) and are completely pinhole-free (Table 5). This is a classic case of thin film metastability. The PVP layer adheres to the AEAPTES substrate via hydrogen bonding between the carbonyl groups in PVP and the amine groups on AEAPTES. The metastability is most likely caused by the disruption of the hydrogen bonding interactions by the hydrophobic moieties on AEAPTES.

**Table 5:**
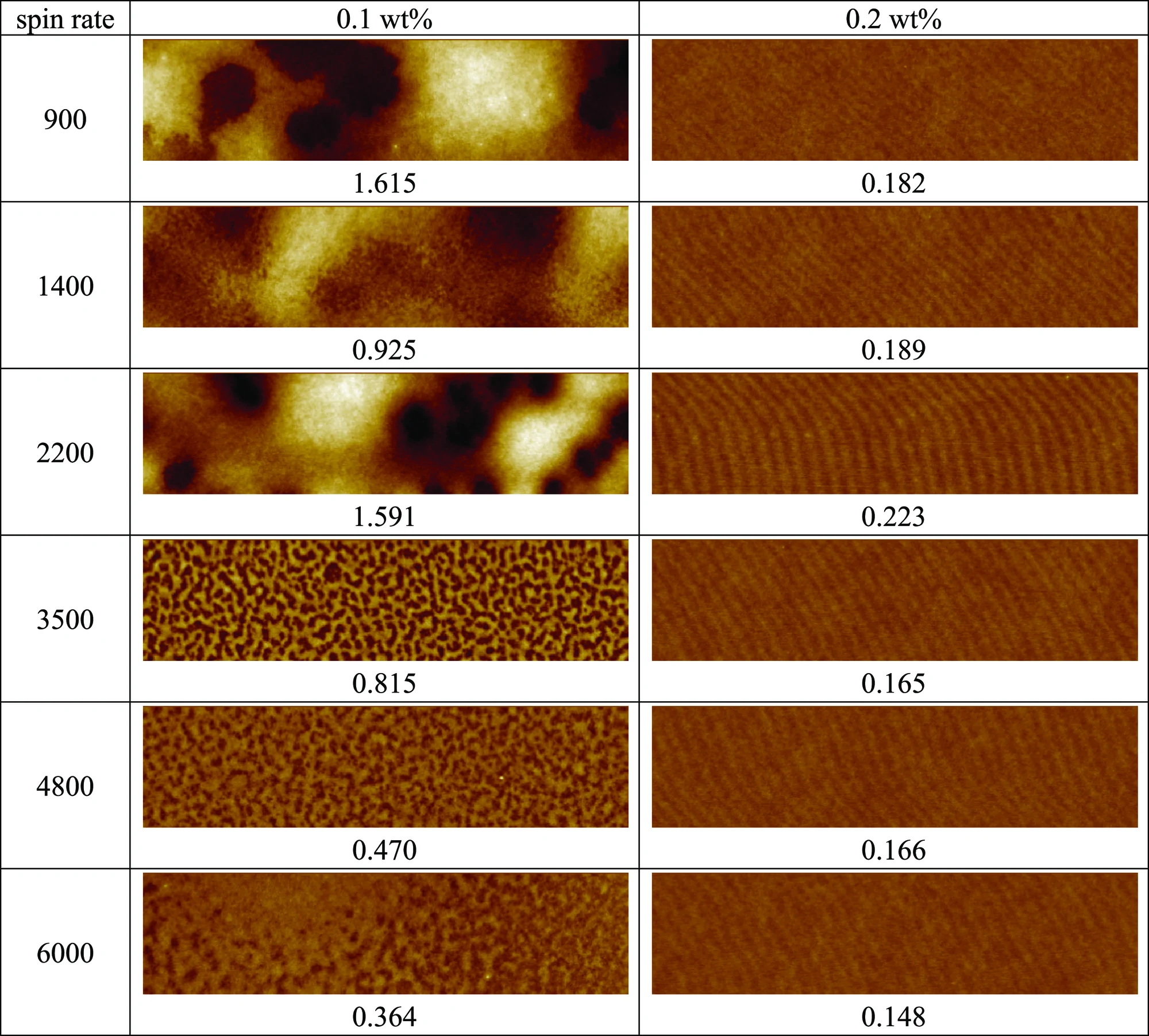
AFM Images (Size: 5 × 1.25 μm^2^; Data Scale: 10 nm) and Root-Mean-Square Roughness (nm) of Spin-Coated PVP Films on AEAPTES as a Function of Spin Rate (rpm) Using 0.1 and 0.2 wt % PVP Solutions

To further explore the effect of substrate hydrophobicity, PVP films were spin-coated on the C8 substrate with 93% alkyl coverage and 7% unreacted silanol groups. Although 0.1 wt % PVP films on C8 exhibit extensive nanoscopic and microscopic dewetting (Tables 6 and 6S), similar to those on TMS, increasing the solution concentration markedly improves film stability. Like AEAPTES, 0.2 wt % PVP films on C8 are substantially thicker and appear largely smooth under AFM (Table 6) and completely smooth under optical microscopy (Table 6S), indicating PVP is also metastable on C8. Contact angle data (Figure 4S), especially advancing contact angles, corroborate the dewetting-to-wetting transition as PVP film thickness increases. Comparing both metastable systems, the extent of dewetting on C8 is more pronounced. Specifically, thin PVP films only dewet nanoscopically on AEAPTES (Tables 5 and 5S), while they dewet both nanoscopically and microscopically on C8. Additionally, the statically adsorbed PVP is continuous on AEAPTES and discontinuous on C8 (Table 2). Together, these observations underscore the destabilizing effect of substrate hydrophobicity on PVP films during the drying process. We further hypothesize the importance of hydrogen bonding in (meta)­stabilizing PVP films on SiO_2_, AEAPTES, and C8.

**Table 6:**
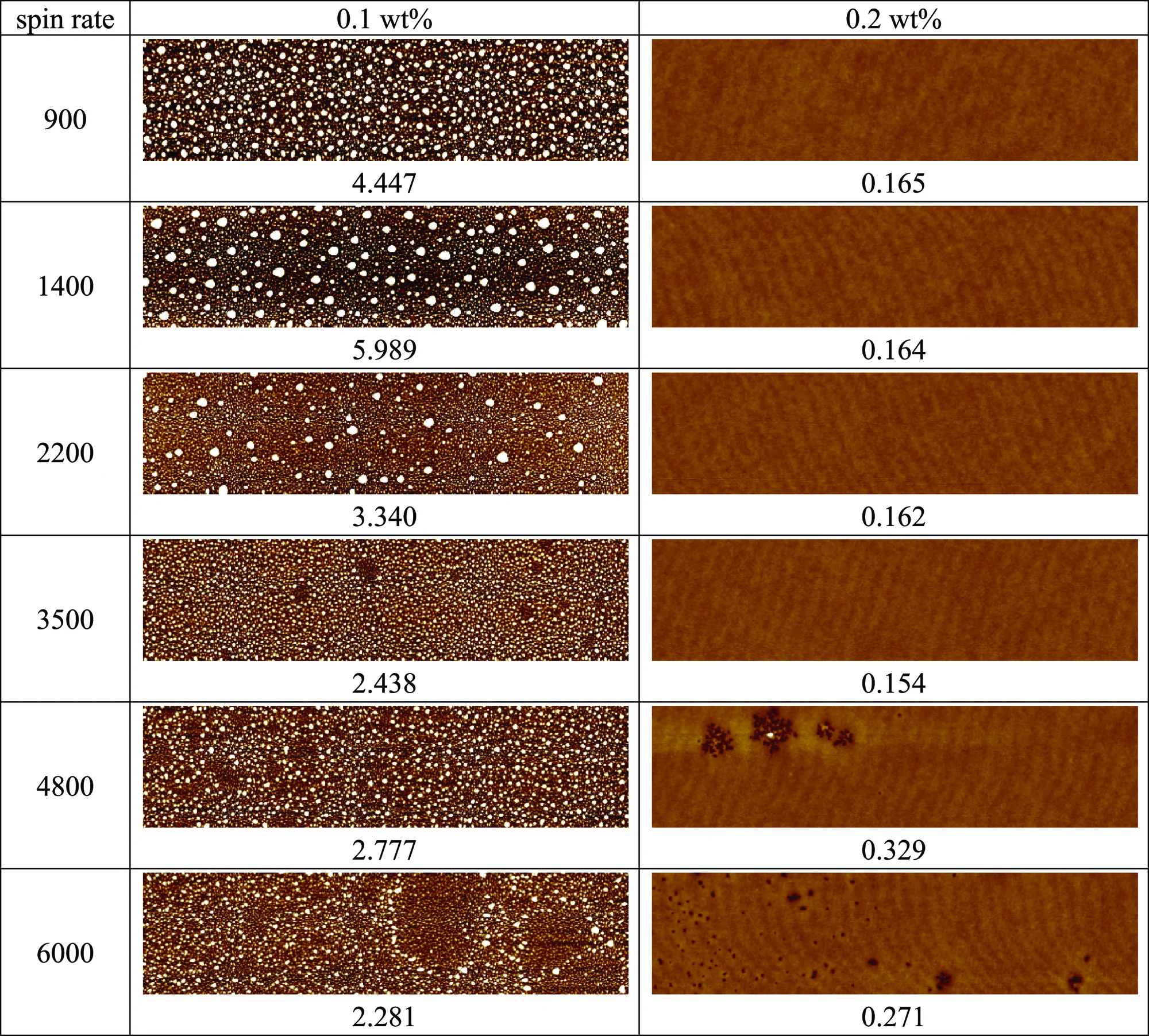
AFM Images (Size: 5 × 1.25 μm^2^; Data Scale: 10 nm) and Root-Mean-Square Roughness (nm) of Spin-Coated PVP Films on C8 as a Function of Spin Rate (rpm) Using 0.1 and 0.2 wt % PVP Solutions

To substantiate this conjecture and bridge the gap between AEAPTES, a mostly hydrophilic substrate, and C8, a mostly hydrophobic substrate, another substrate with a comparable split between hydrophobic groups and hydrogen bonding sites was included in the study. C8 (15 min) was prepared by reducing the reaction time between C8 silane and silicon wafers from 24 h to 15 min. Based on its advancing and receding contact angles of 74 ± 3°/38 ± 3°, surface coverage of C8 (15 min) is ∼61% of the hydrophobic octyl groups and ∼39% of the −OH groups. Spin-coated PVP films on C8 (15 min) are metastable based on the AFM and optical images (Tables 7S and 8S). These films are similar to those on AEAPTES in that only nanoscopic dewetting takes place in thin PVP films. Such results underscore the necessity of having a threshold amount of hydrogen bonding sites on a substrate to prevent microscopic dewetting and stabilize the spin-deposited PVP layers.

## Conclusions

Four substrates (SiO_2_, AEAPTES, C8, and TMS) with different proportions of hydrogen bonding sites and hydrophobic groups were successfully fabricated to study PVP adsorption and film stability. Both hydrogen bonding and hydrophobic interactions drive PVP adsorption at the substrate–solution interface. This work demonstrated that PVP is a universal adhesive in that it spontaneously adheres to all the substrates, forming 1 nm-thick films. However, the PVP film continuity and stability are substantially substrate-dependent. The presence of hydrophobic groups increases substrate-PVP interfacial free energy and can induce film rupture during drying. Through judicious substrate-chemistry control and imaging studies, this study established that hydrogen bonding is required to pin PVP chains at the interface and impart film (meta)­stability. These findings provide molecular-level design principles for developing robust hydrophilic polymer coatings.

## Supplementary Material



## Notes

Additional AFM images; optical images; and contact angle data of the four substrates and PVP thin films on the substrates (PDF)
