# Supplementary material for: Hydrogen Bonding is Required for Continuous Polyvinylpyrrolidone Adhesive Films
Source: Langmuir. 2026 Oct 8;42(41):31061–9. doi: 10.1021/acs.langmuir.6c04951 (PMC13689932; doi:10.1021/acs.langmuir.6c04951)
Supplement: Supplementary file 1 [file la6c04951_si_001.pdf]

**Hydrogen bonding is required for continuous polyvinylpyrrolidone adhesive films**

Mahira Mim, An C. N. Nguyen, Mamie Mulder, Hanae Takahashi, Mackenzie Windus, and Wei Chen*

*To whom correspondence should be addressed
ORCID Wei Chen: 0000-0002-6970-3455
Email: weichen@mtholyoke.edu

Address: Chemistry Department, Carr Laboratory, Mount Holyoke College, 50 College Street, South Hadley, Massachusetts 01075

Pages: 12
Figures: 4
Tables: 8

**Table 1S:** AFM images (size: 5 µm x 1.25 µm; data scale: 10 nm) of different substrates.

| $SiO_2$ | TMS | C8 | AEAPTES |
|---|---|---|---|
| 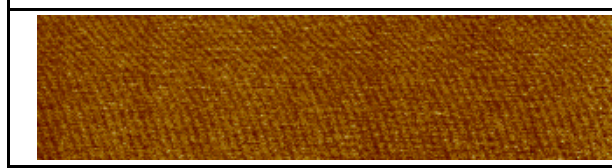 | 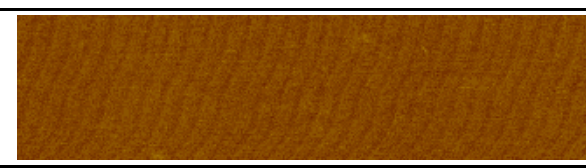 | 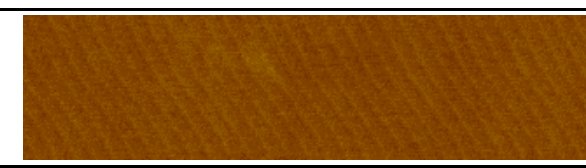 | 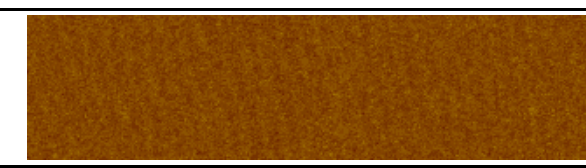 |

**Table 2S**. AFM Images (size: 5 µm x 1.25 µm; data scale: 10 nm) and optical images (size: 362 µm x 226 µm) of spin-coated PVP films on TMS using 0.2 wt% PVP solution.

| spin rate | AFM | optical |
|---|---|---|
| 900 | 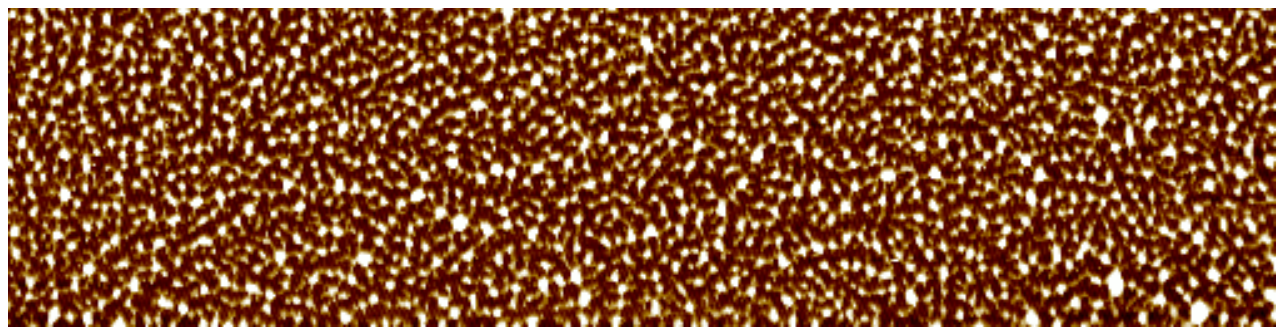 | 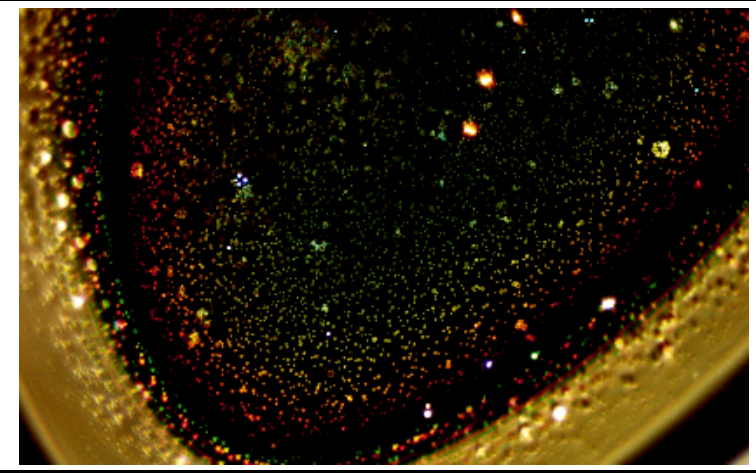 |
| 1400 | 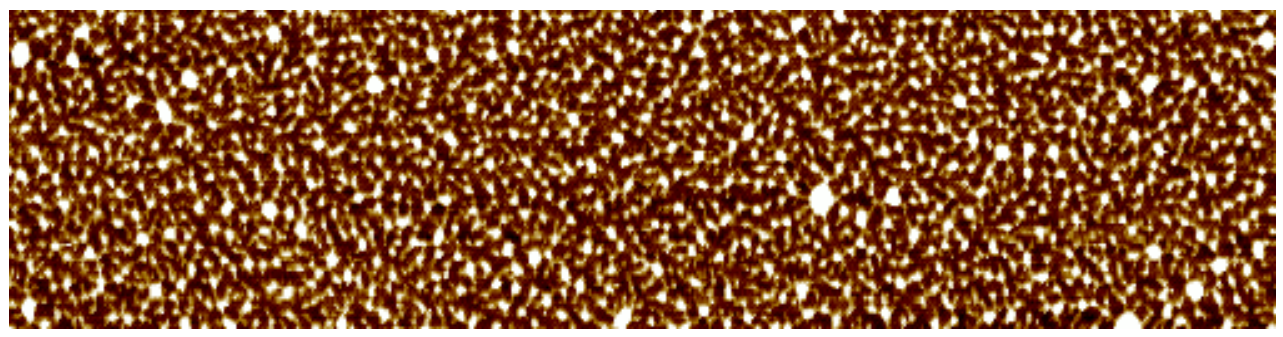 | 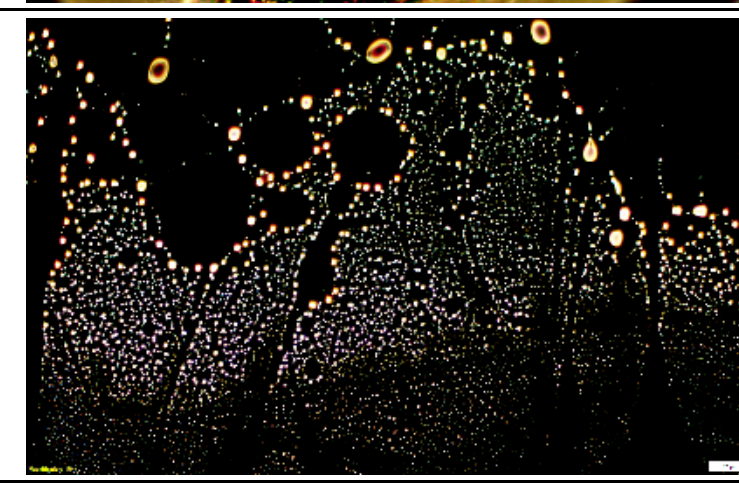 |
| 2200 | 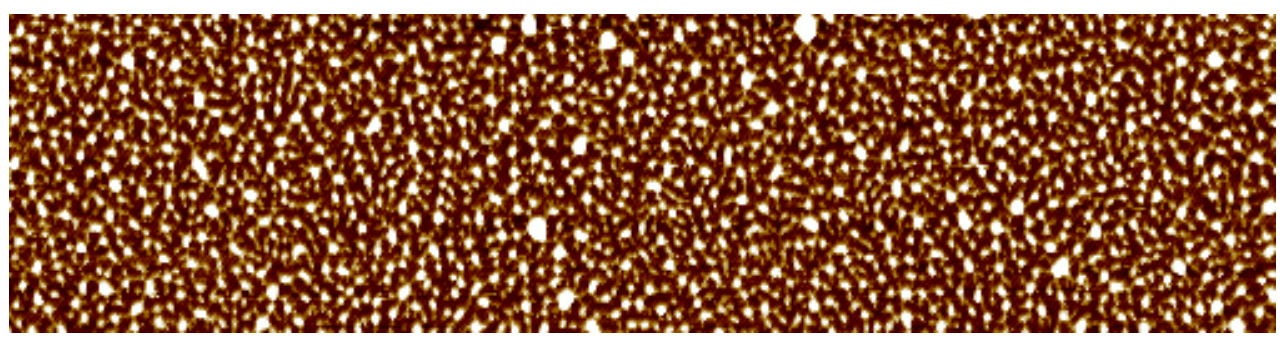 | 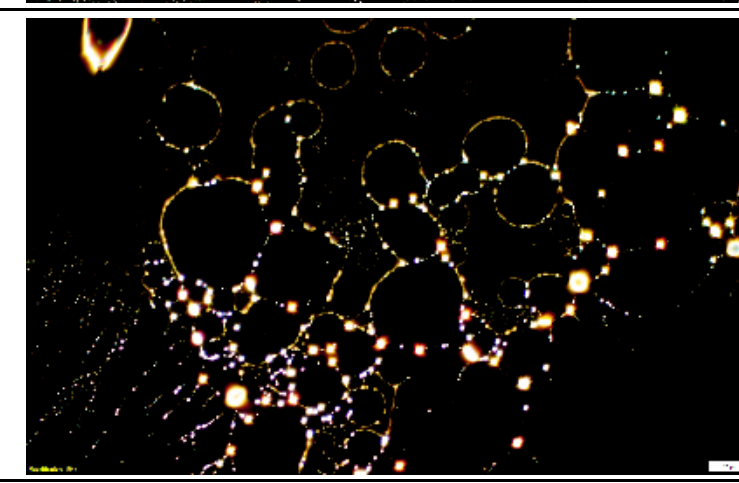 |
| 3500 | 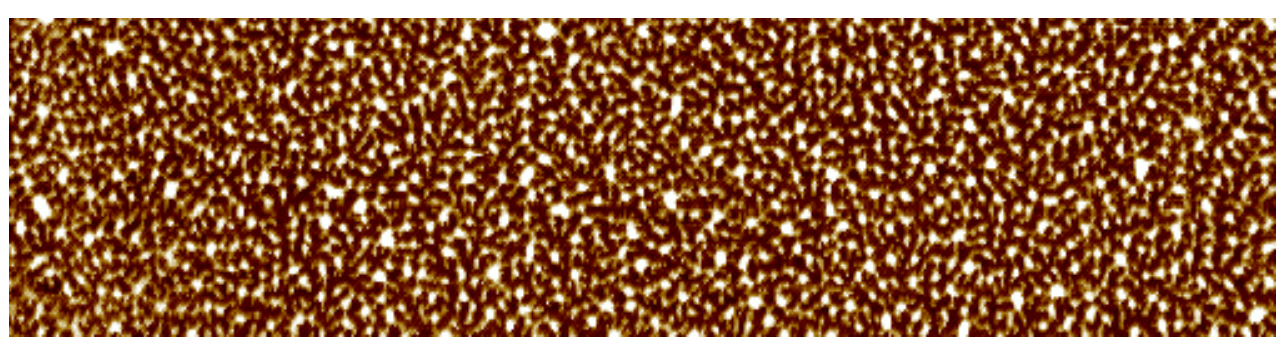 | 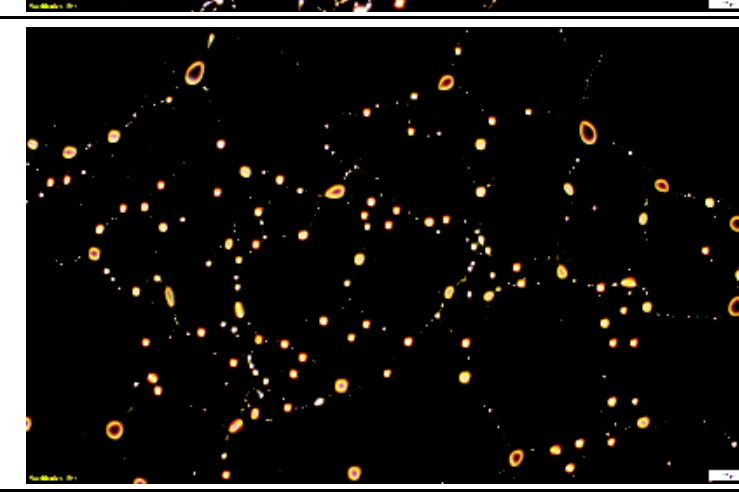 |
| 4800 | 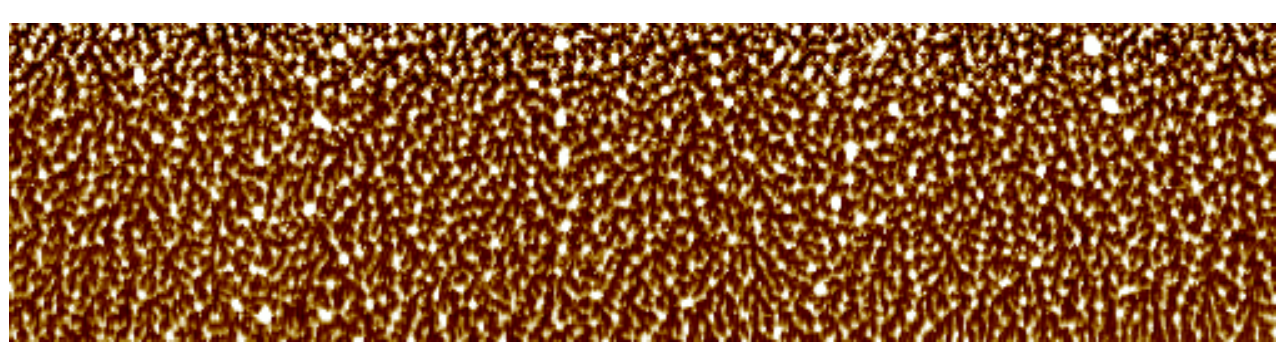 | 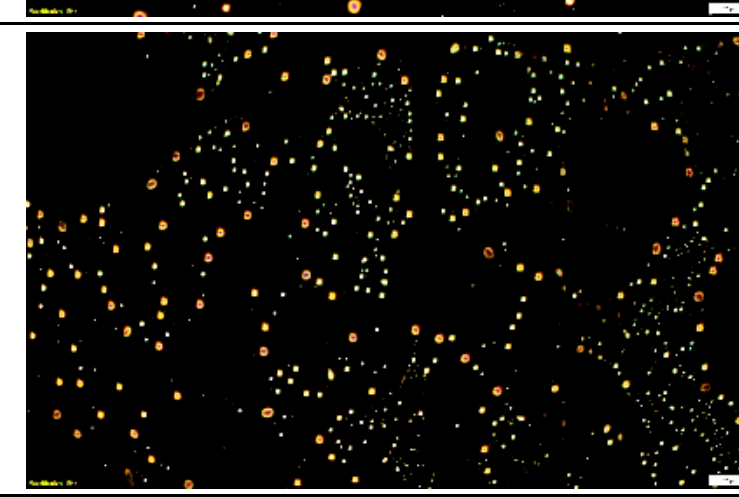 |
| 6000 | 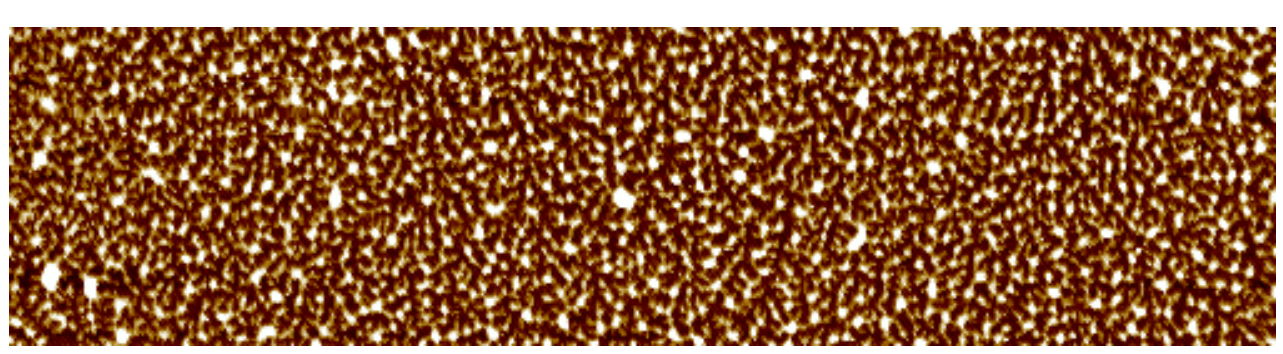 | 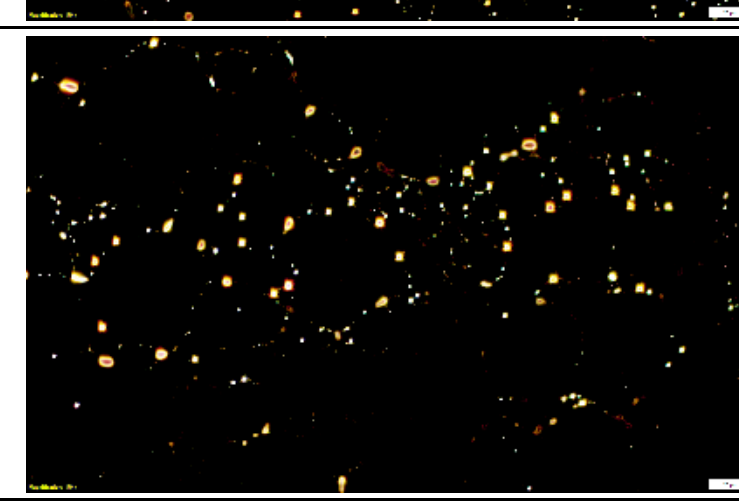 |

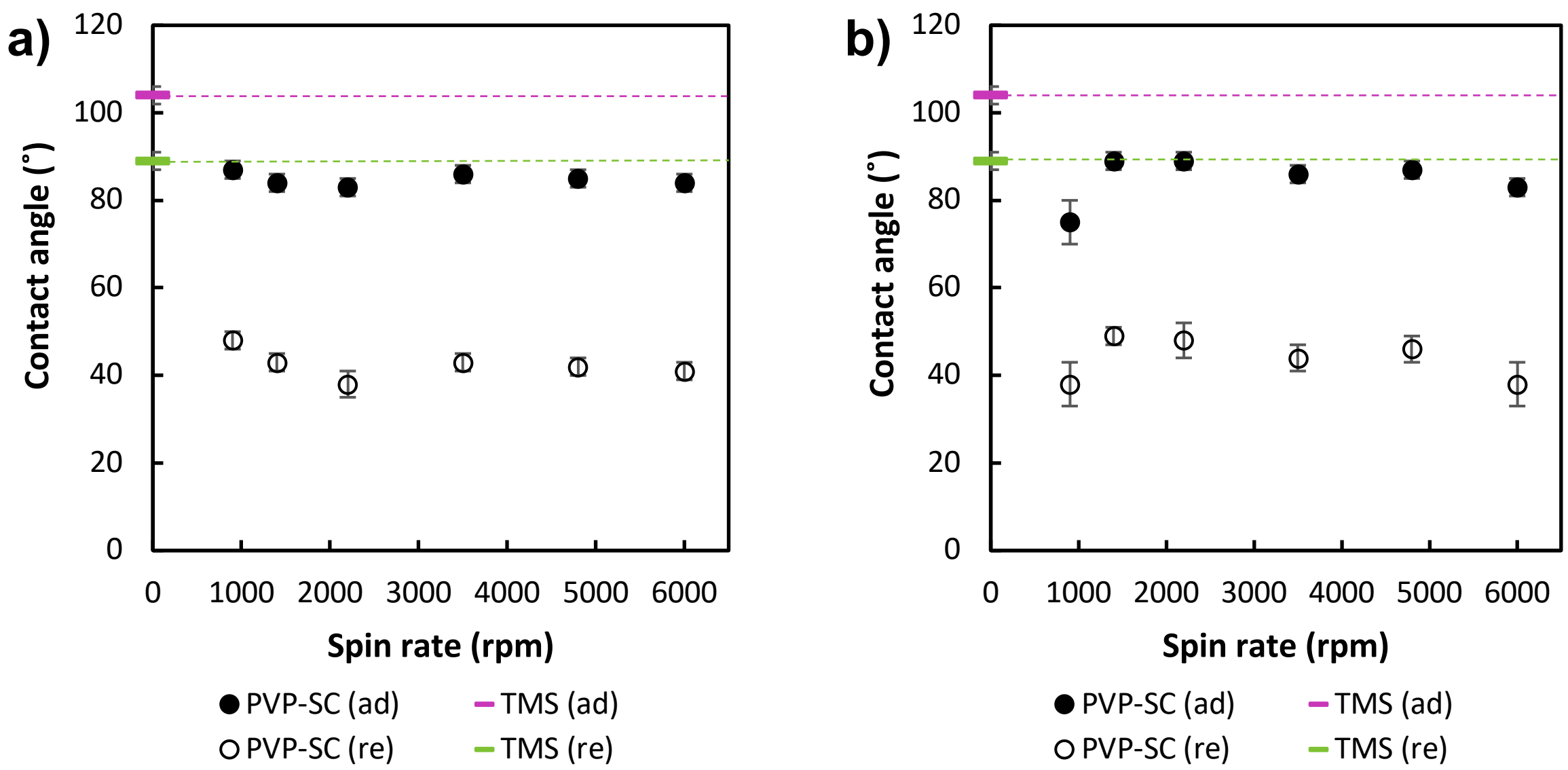


**Figure 1S.** Advancing (ad) and receding (re) water contact angles (°) of spin-coated PVP films (PVP-SC) on TMS as a function of spin rate (rpm) using a) 0.1 wt% and b) 0.2 wt% PVP solutions. Straight lines correspond to advancing and receding contact angles of TMS substrate.

**Table 3S.** AFM images (size: 5 µm x 1.25 µm; data scale: 10 nm) and optical images (size: 362 µm x 226 µm) of spin-coated PVP films on $SiO_2$ using 0.2 wt% PVP solution.

| spin rate | AFM | optical |
|---|---|---|
| 900 | 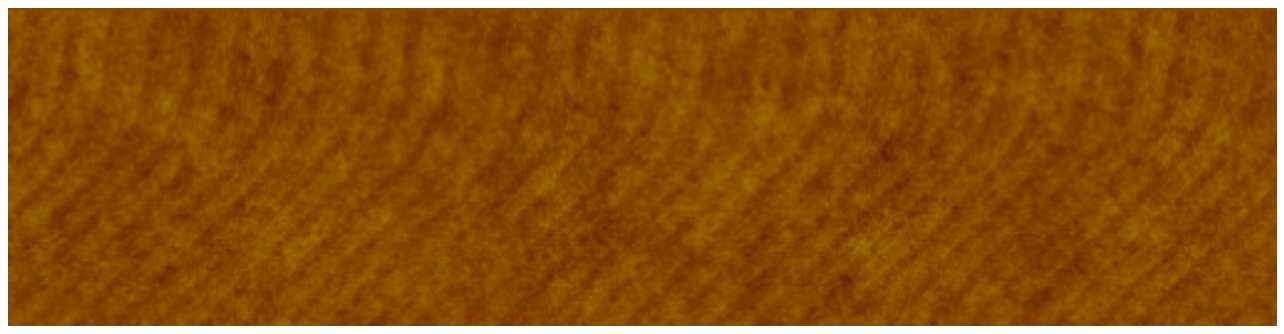 | 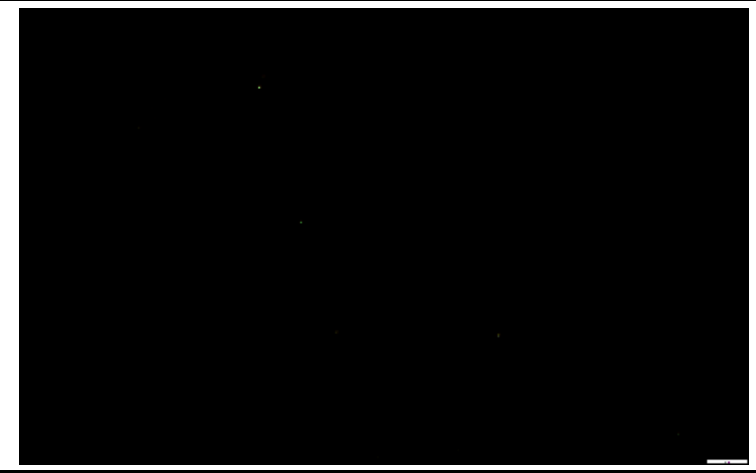 |
| 1400 | 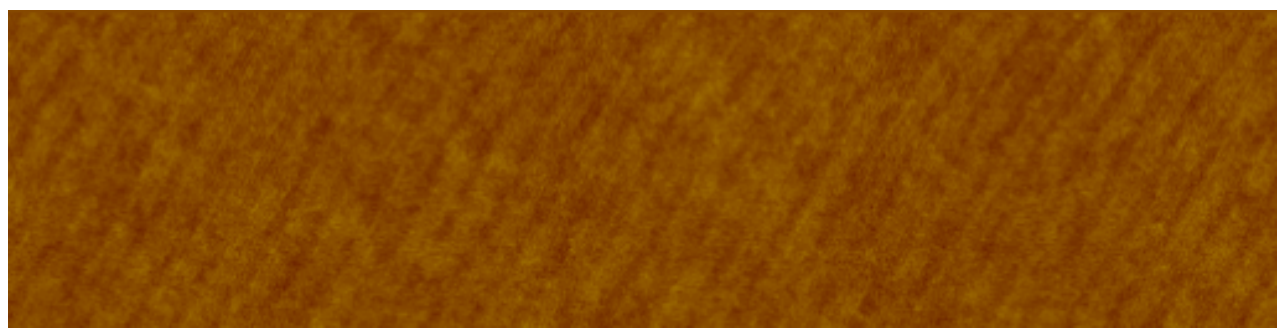 | 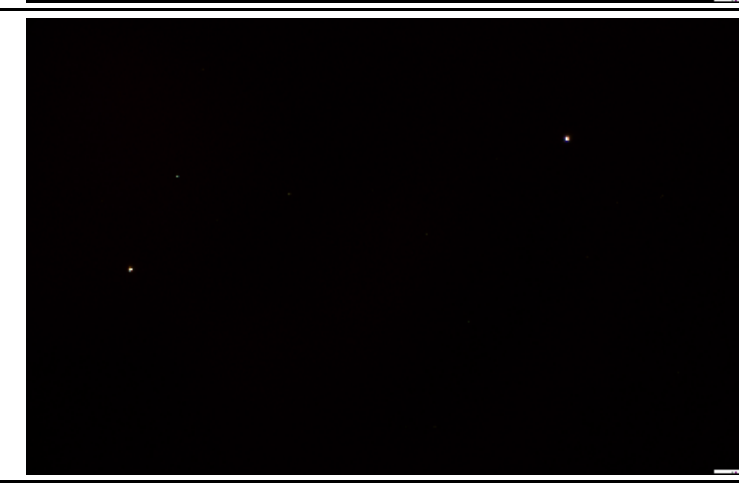 |
| 2200 | 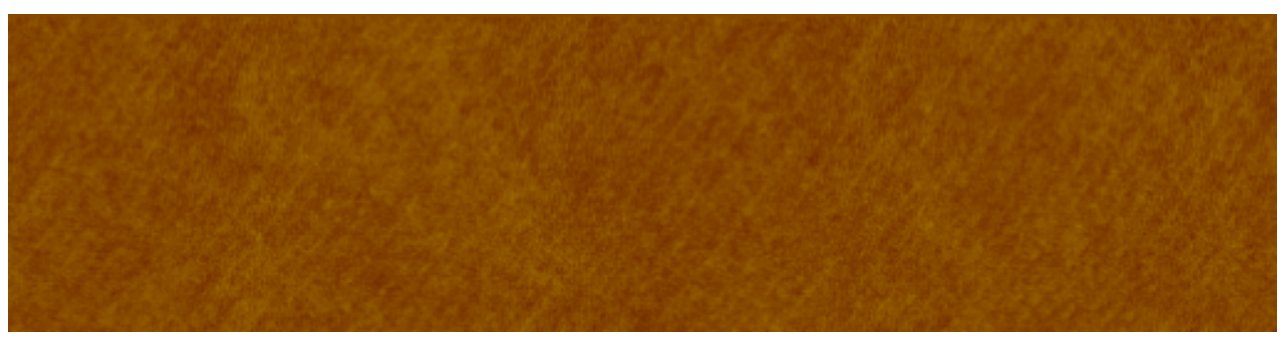 | 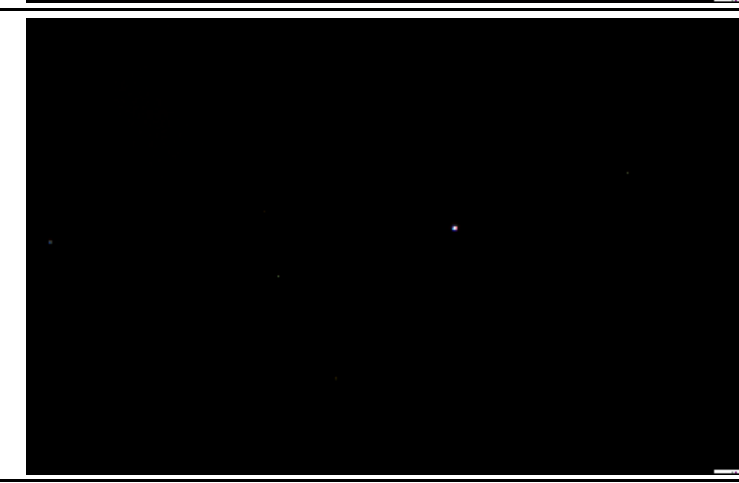 |
| 3500 | 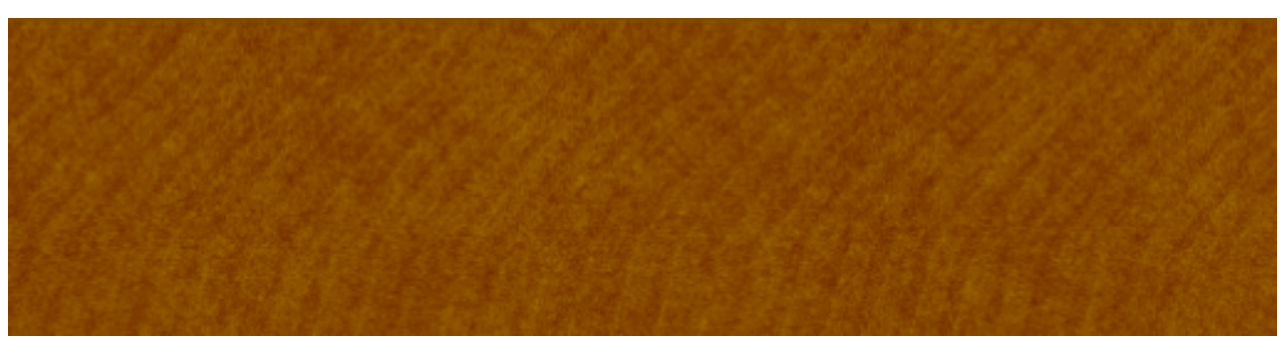 | 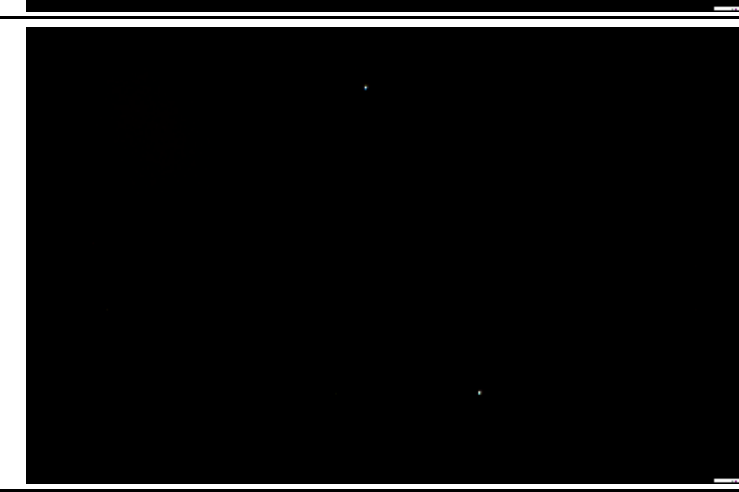 |
| 4800 | 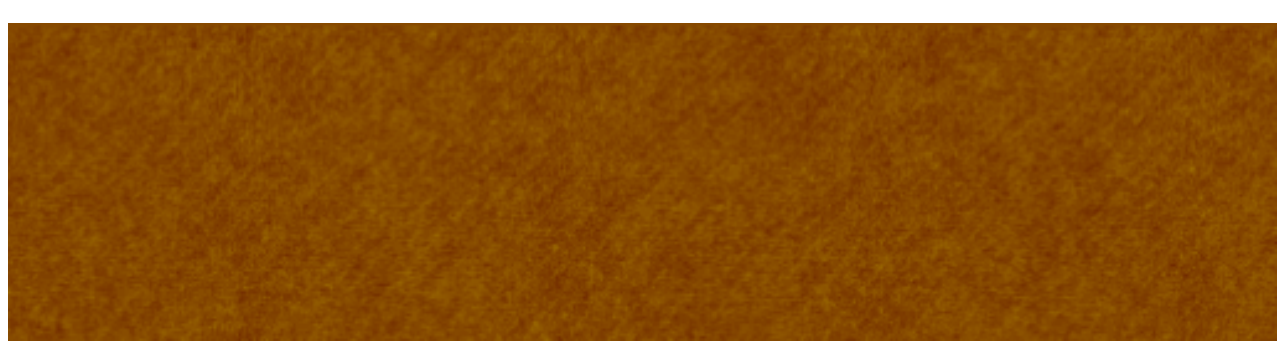 | 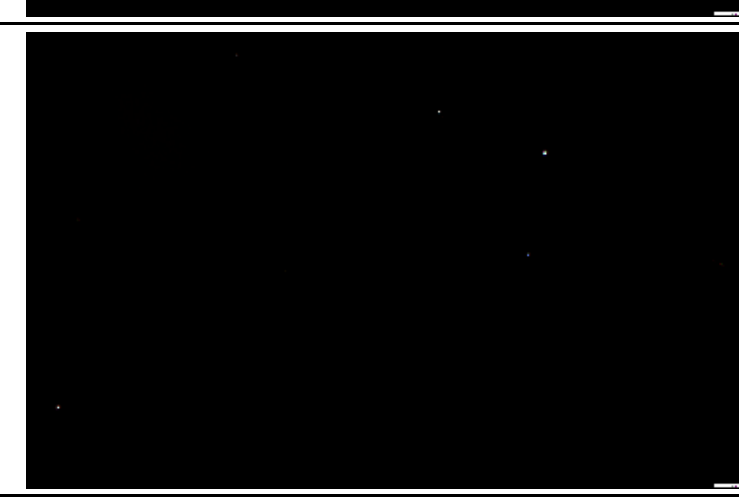 |
| 6000 | 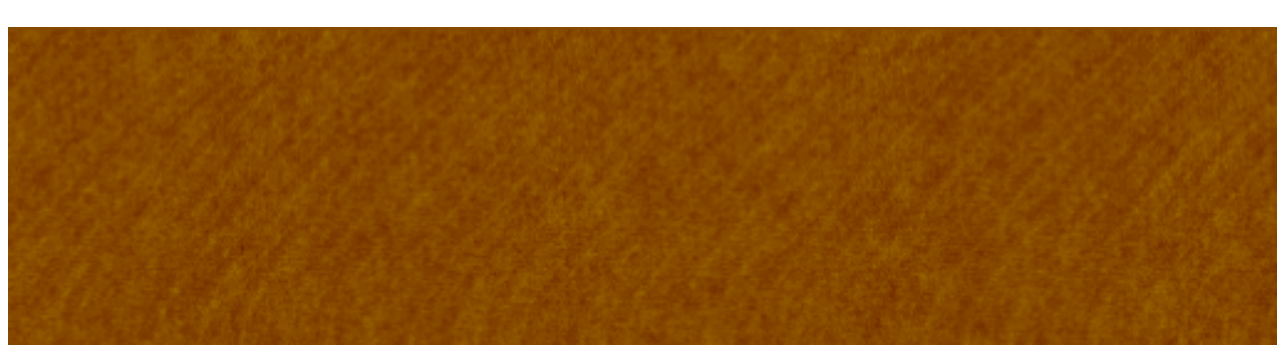 | 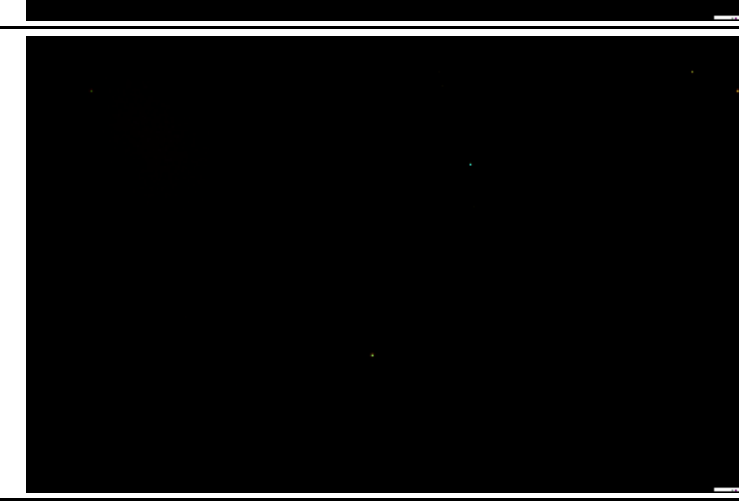 |

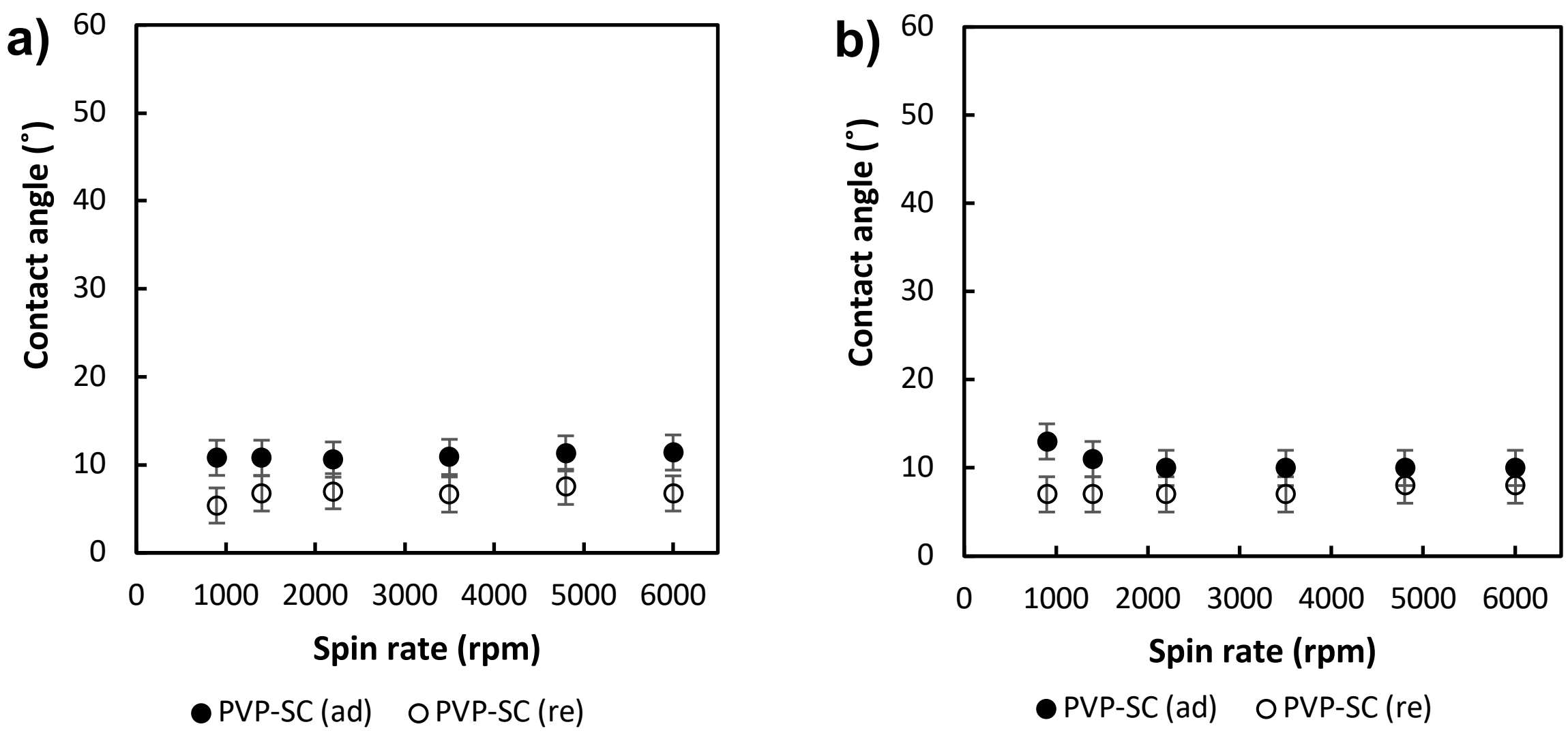


**Figure 2S.** Advancing (ad) and receding (re) water contact angles (°) of spin-coated PVP films (PVP-SC) on $SiO_2$ as a function of spin rate (rpm) using a) 0.1 wt% and b) 0.2 wt% PVP solutions.

**Table 4S.** AFM Images (size: 5 x 1.25 µm; data scale: 5 nm) of spin-coated PVP films prepared on $SiO_2$ at 6000 rpm from 0.1 wt% solution: before, after thermal annealing (T. A.) at 150 °C for 15 min without water, and after thermal annealing at 150 °C for 15 min with 20 µL of water (no direct contact).

| Before T. A. | After T. A. | |
|---|---|---|
| | without water | with water |
| 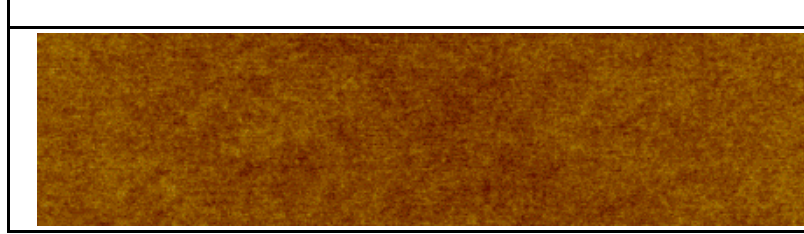 | 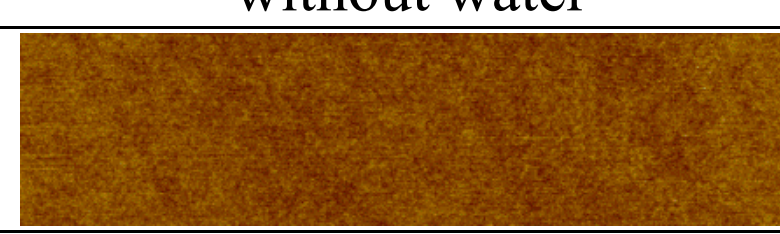 | 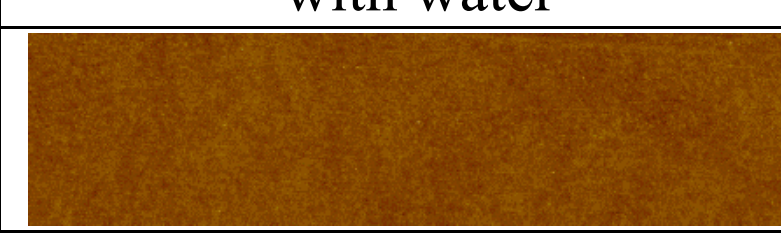 |

**Table 5S.** Optical images (size: 362 μm x 226 μm) of spin-coated PVP films on AEAPTES using 0.1 wt% and 0.2 wt% PVP solutions.

| spin rate | 0.1 wt% | 0.2 wt% |
|---|---|---|
| 900 | 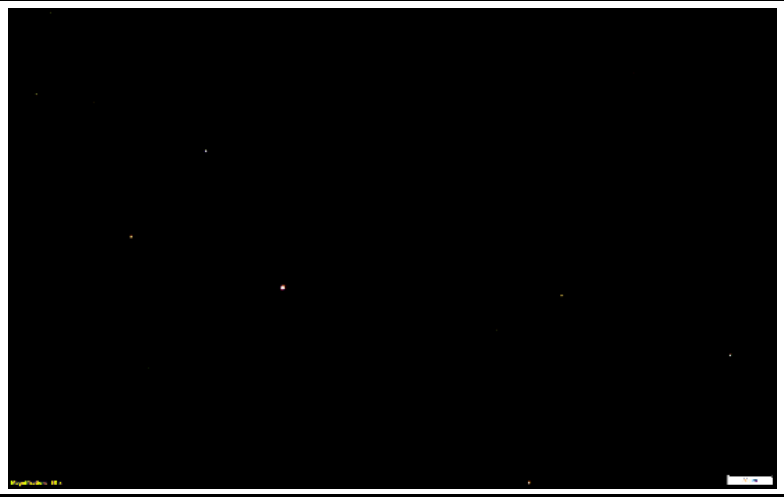 | 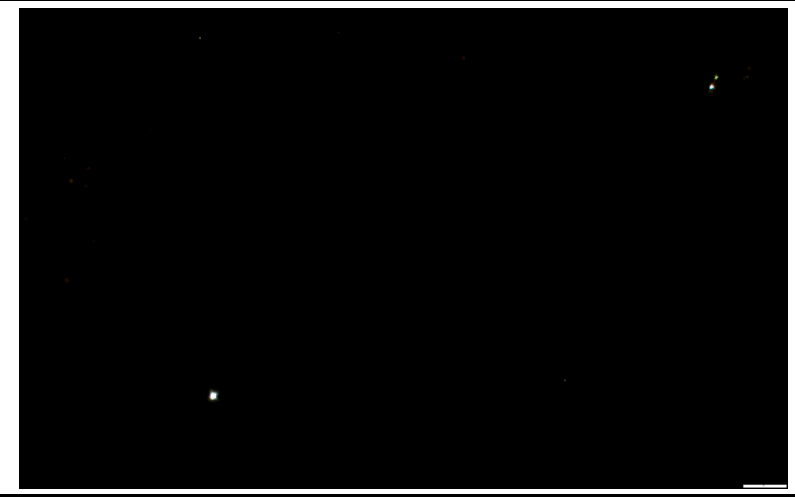 |
| 1400 | 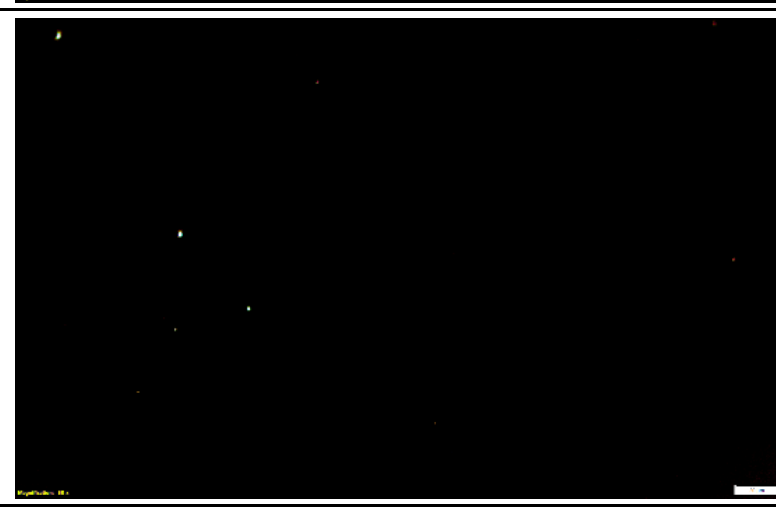 | 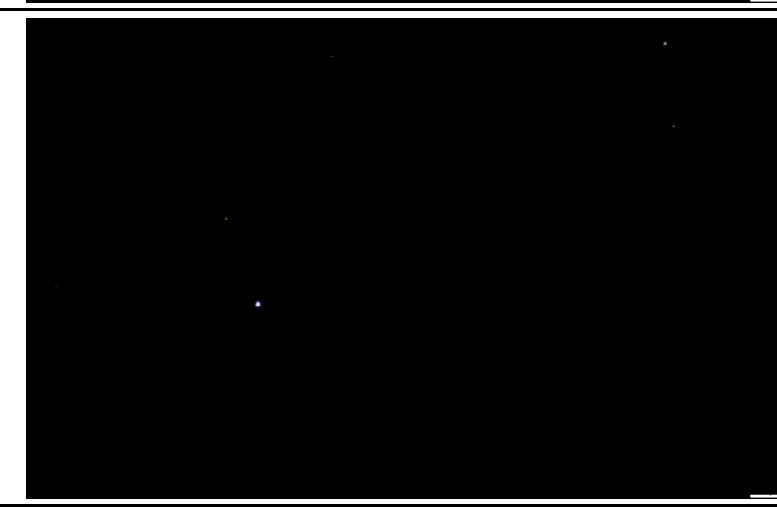 |
| 2200 | 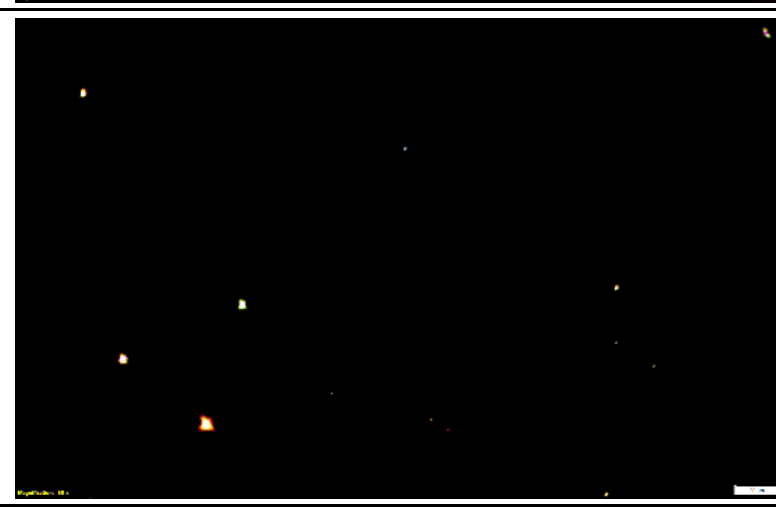 | 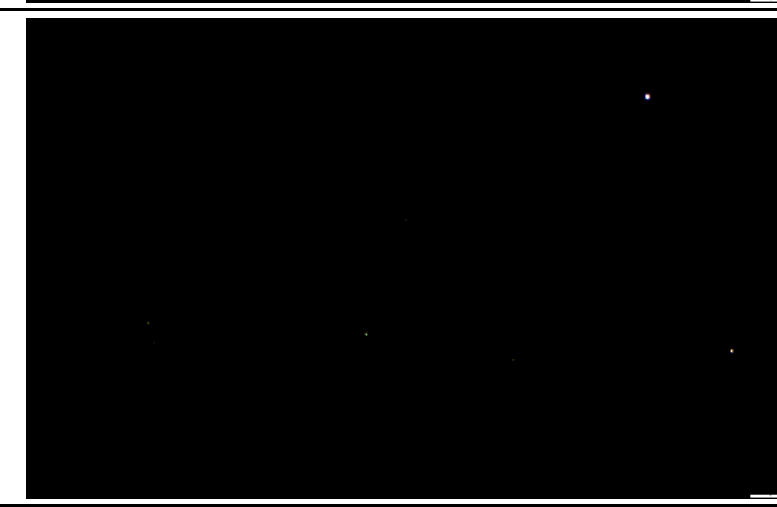 |
| 3500 | 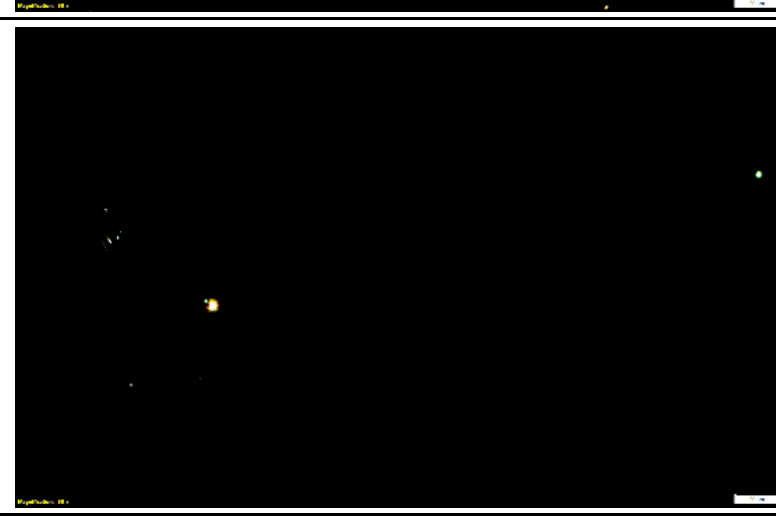 | 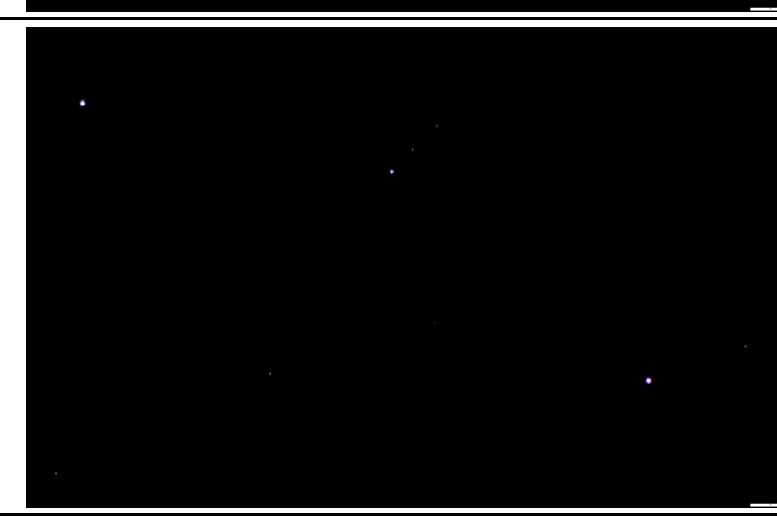 |
| 4800 | 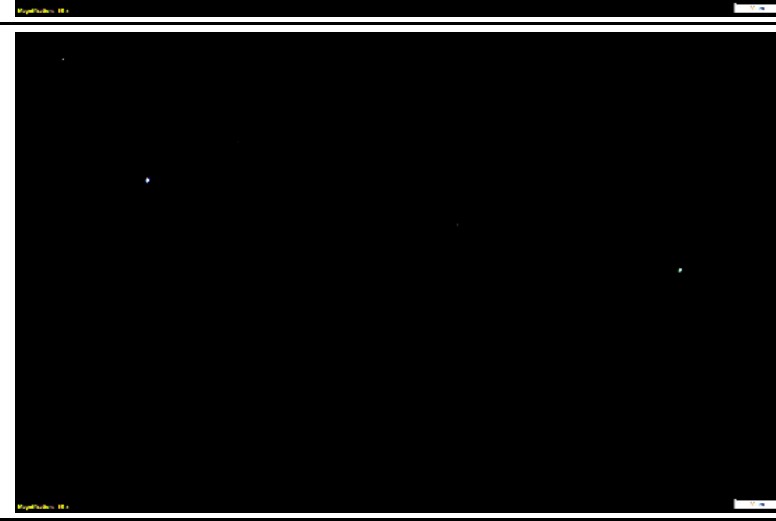 | 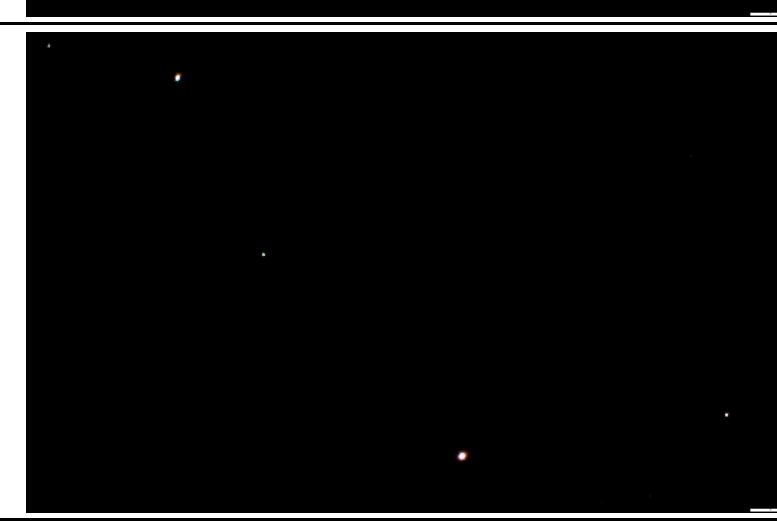 |
| 6000 | 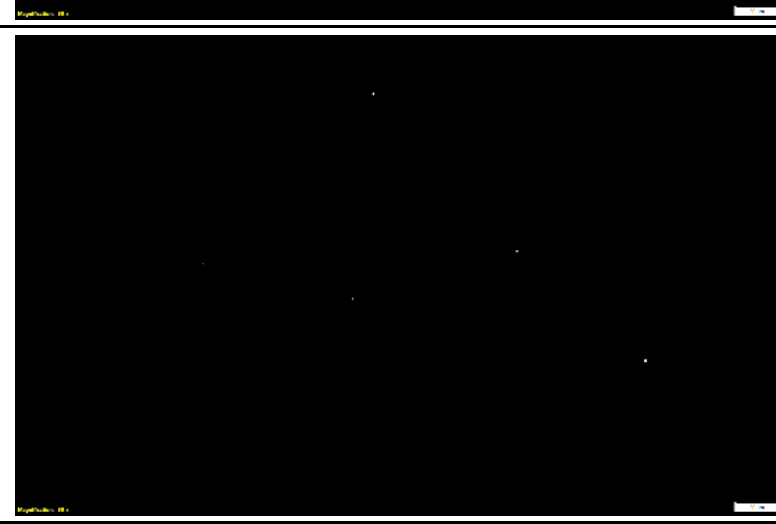 | 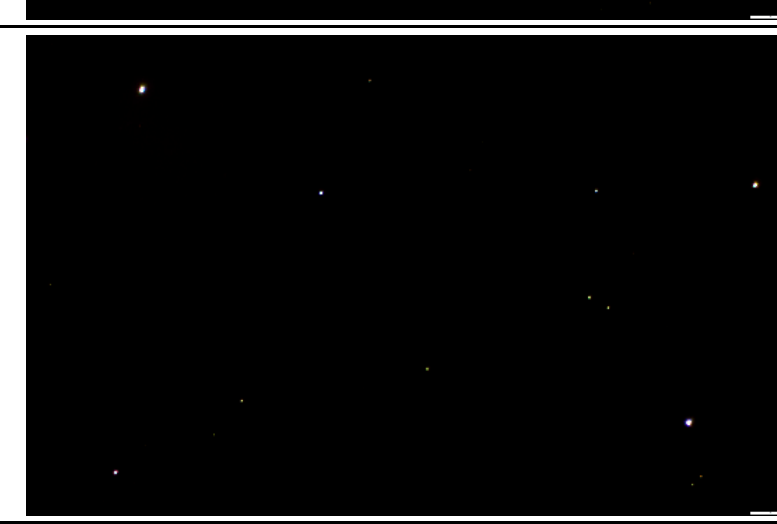 |

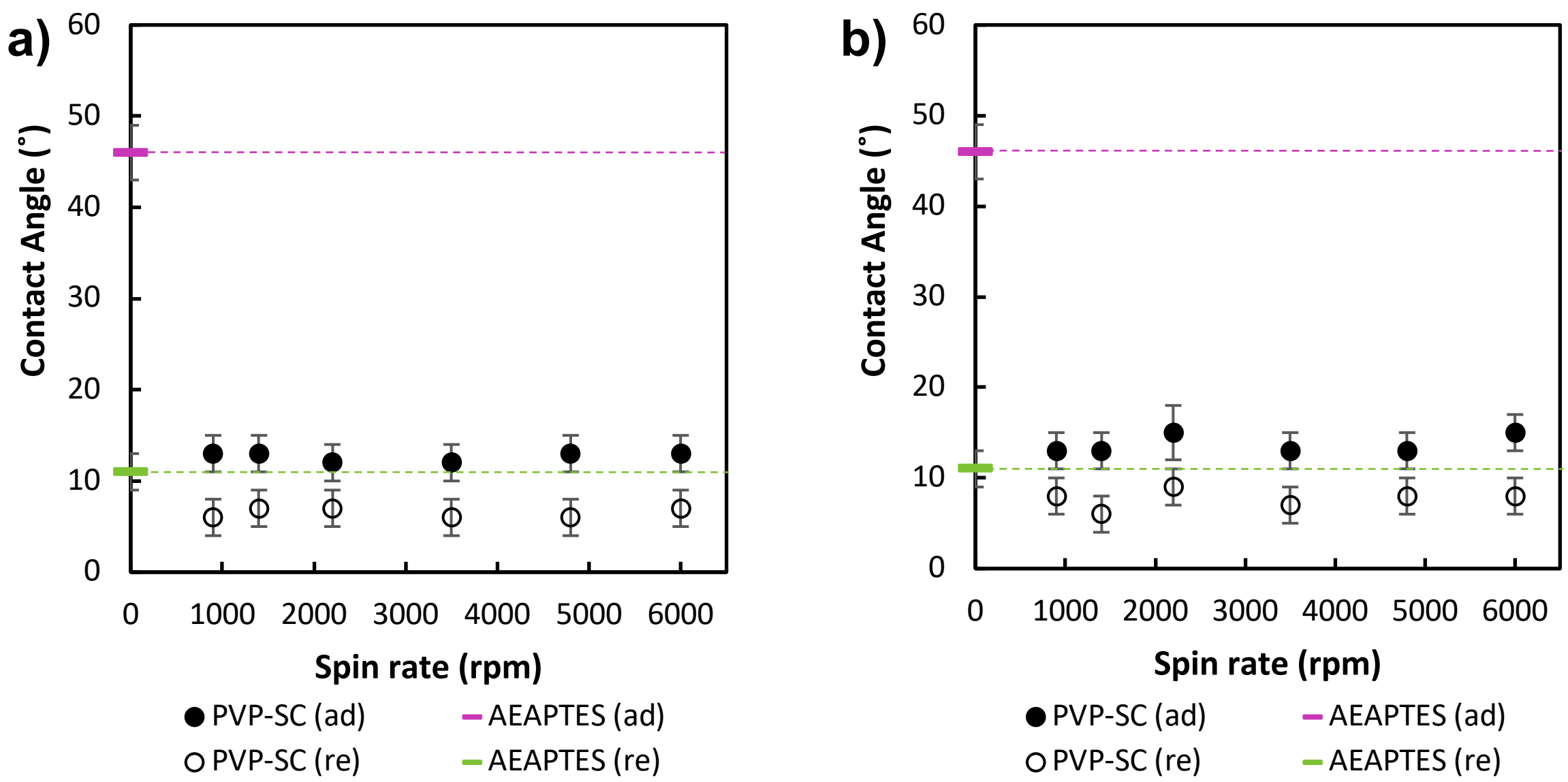


**Figure 3S.** Advancing (ad) and receding (re) water contact angles (°) of spin-coated PVP films (PVP-SC) on AEAPTES as a function of spin rate (rpm) using a) 0.1 wt% and b) 0.2 wt% PVP solutions. Straight lines correspond to advancing and receding contact angles of AEAPTES substrate.

**Table 6S.** Optical images (size: 362 μm x 226 μm) of spin-coated PVP films on C8 using 0.1 wt% and 0.2 wt% PVP solutions.

| spin rate | 0.1 wt% | 0.2 wt% |
|---|---|---|
| 900 | 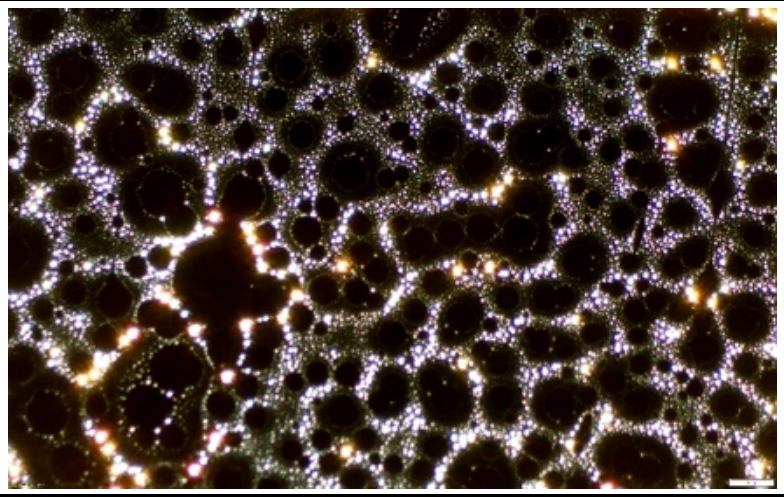 | 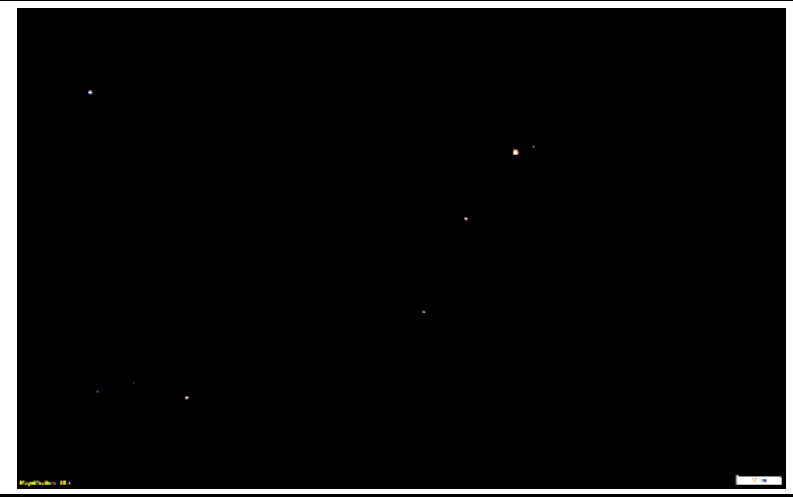 |
| 1400 | 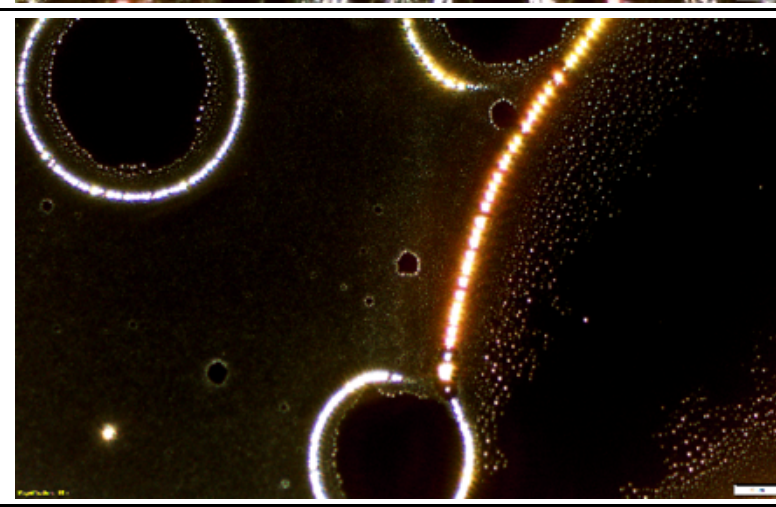 | 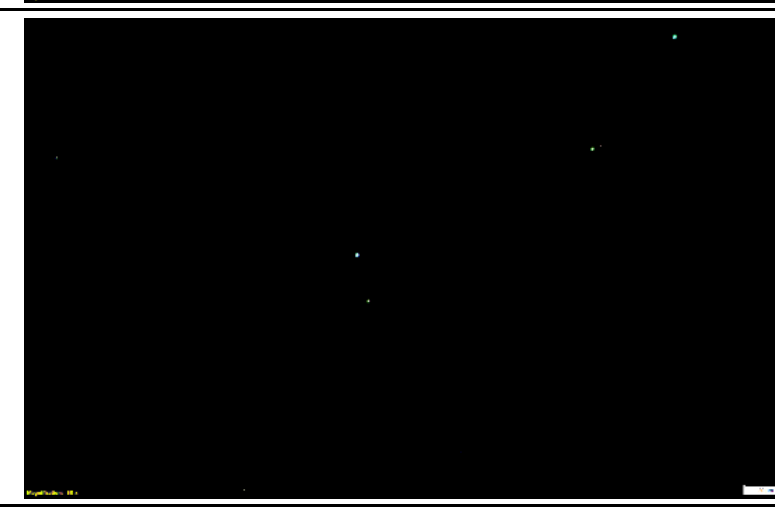 |
| 2200 | 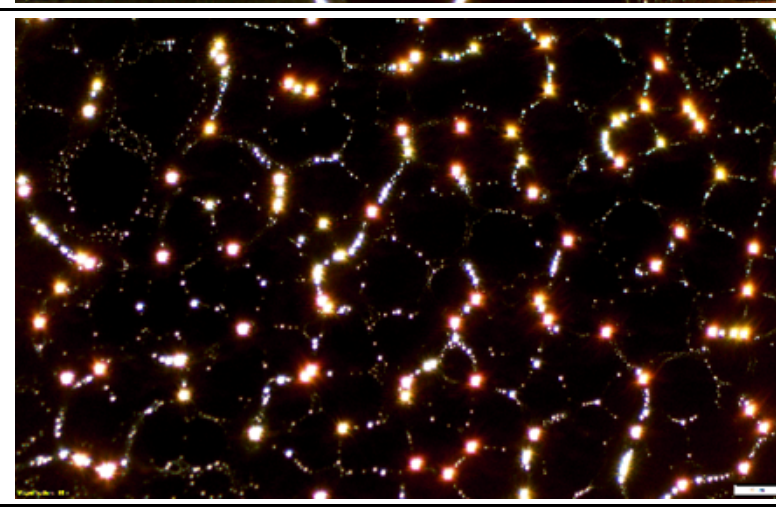 | 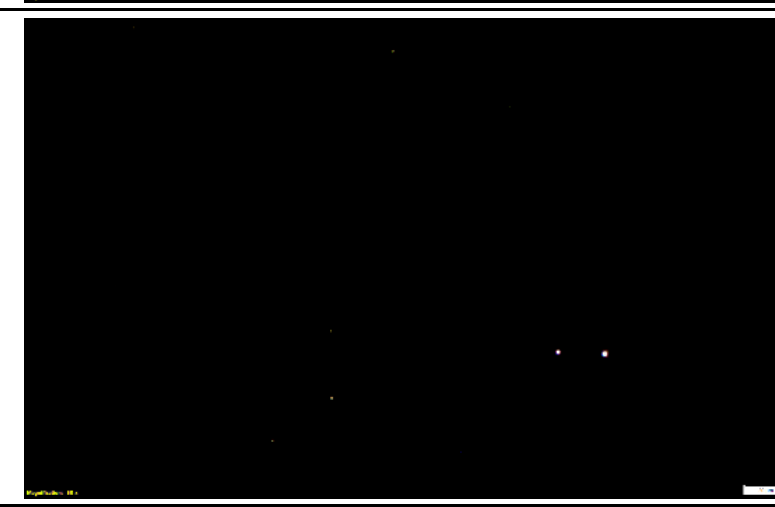 |
| 3500 | 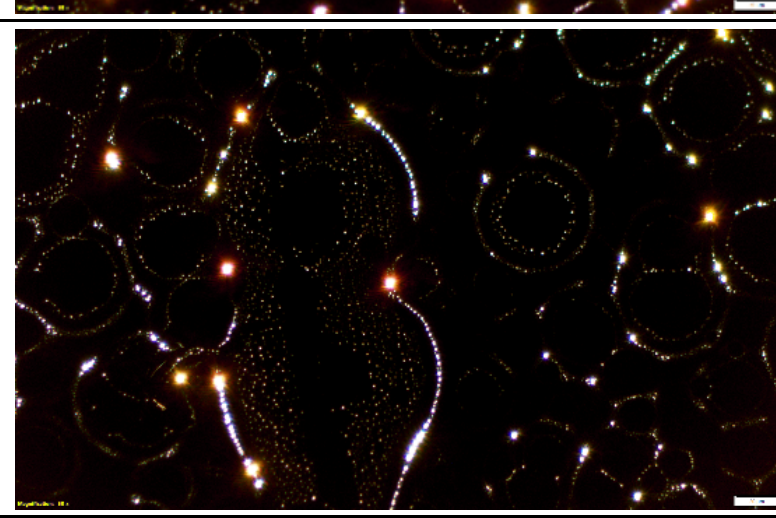 | 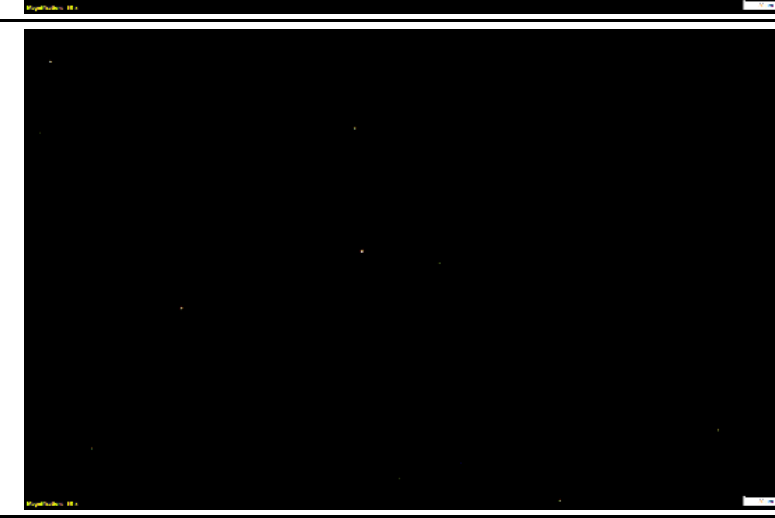 |
| 4800 | 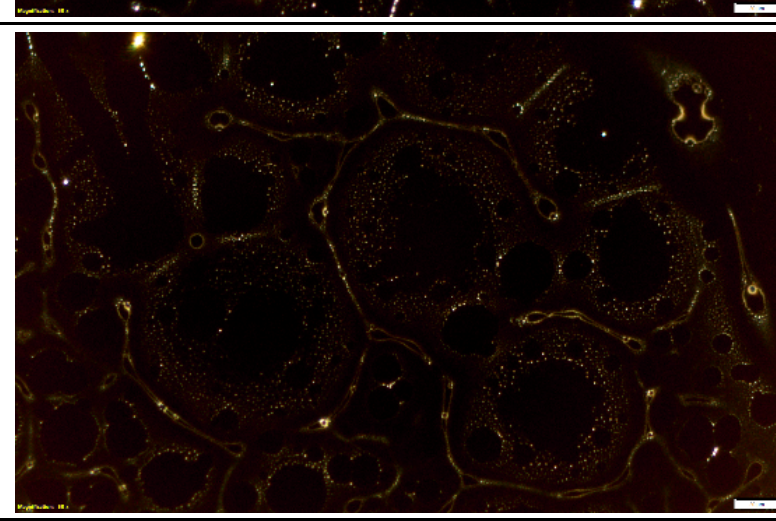 | 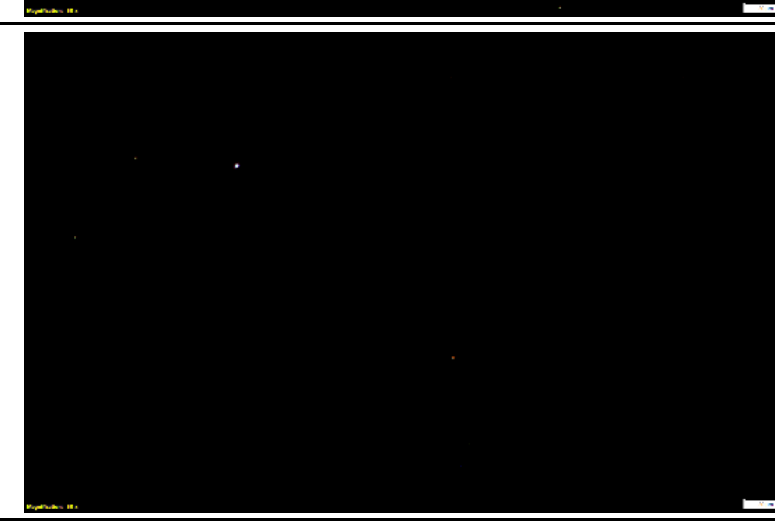 |
| 6000 | 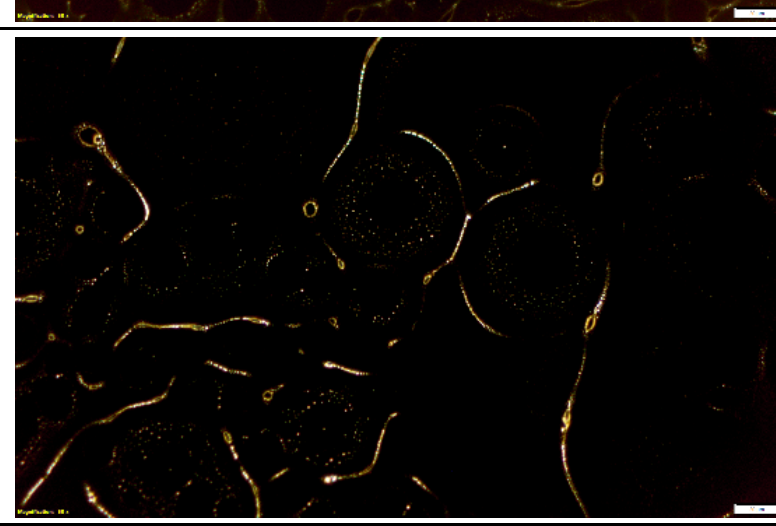 | 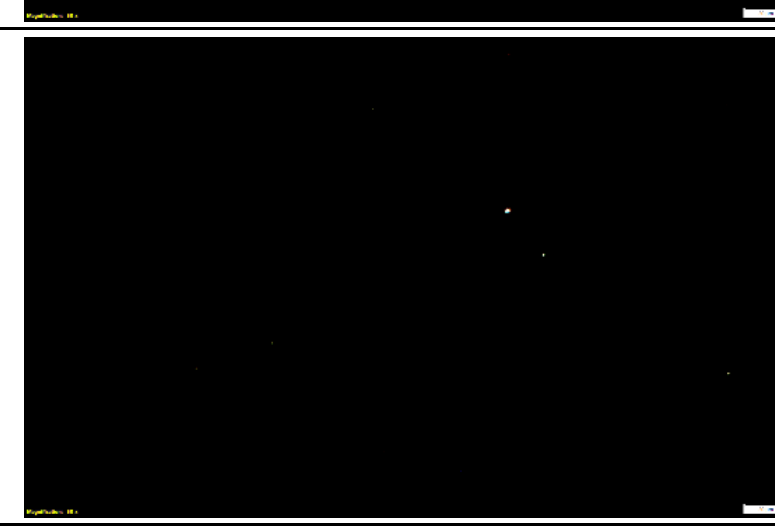 |

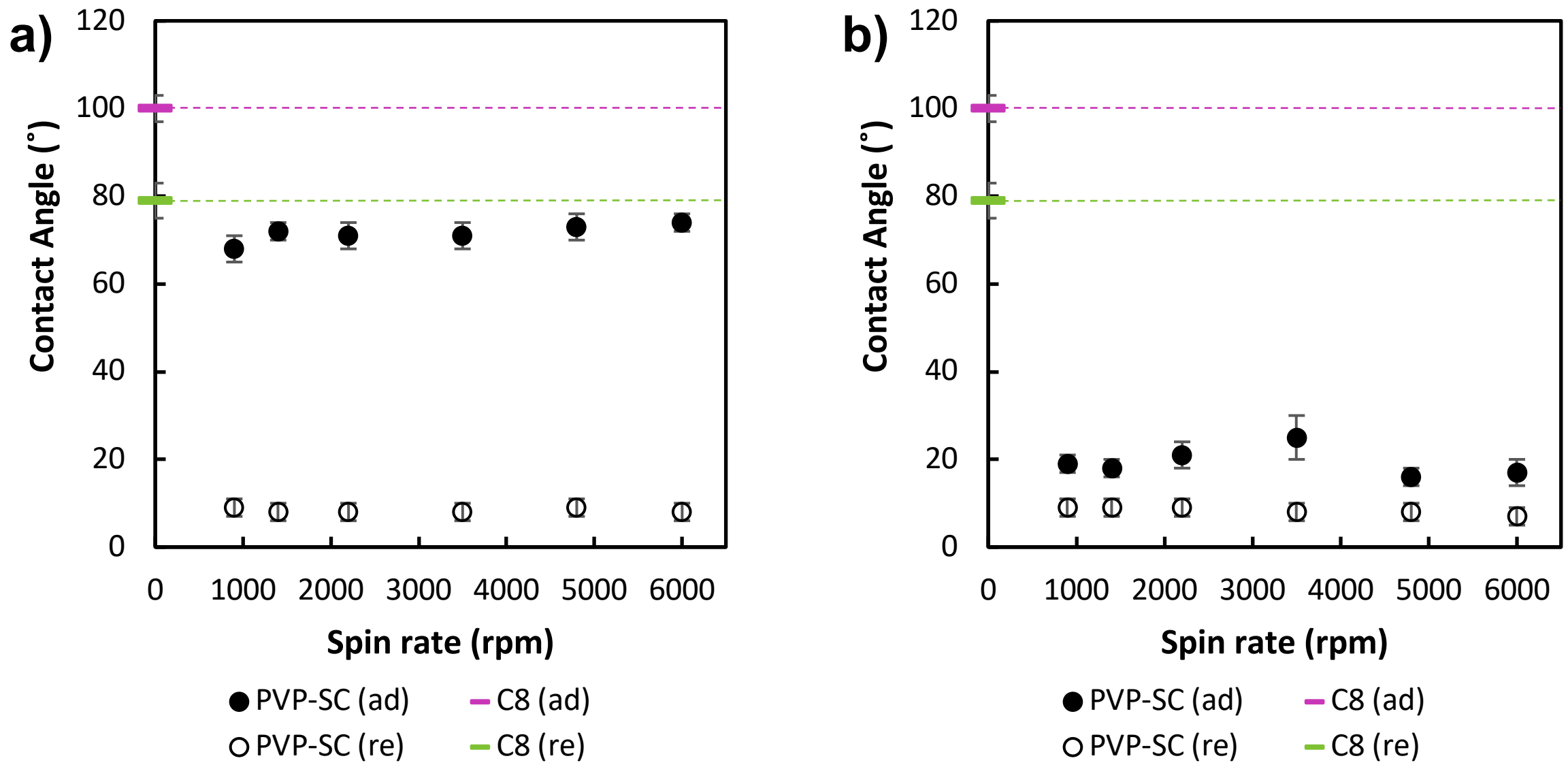


**Figure 4S.** Advancing (ad) and receding (re) water contact angles (°) of spin-coated PVP films (PVP-SC) on C8 as a function of spin rate (rpm) using a) 0.1 wt% and b) 0.2 wt% PVP solutions. Straight lines correspond to advancing and receding contact angles of C8 substrate.

**Table 7S.** AFM Images (size: 5 x 1.25 µm; data scale: 10 nm) and optical images (size: 362 µm x 226 µm) of spin-coated 0.1 wt% PVP on C8 (15 min).

| spin rate | AFM | optical |
|---|---|---|
| 900 | 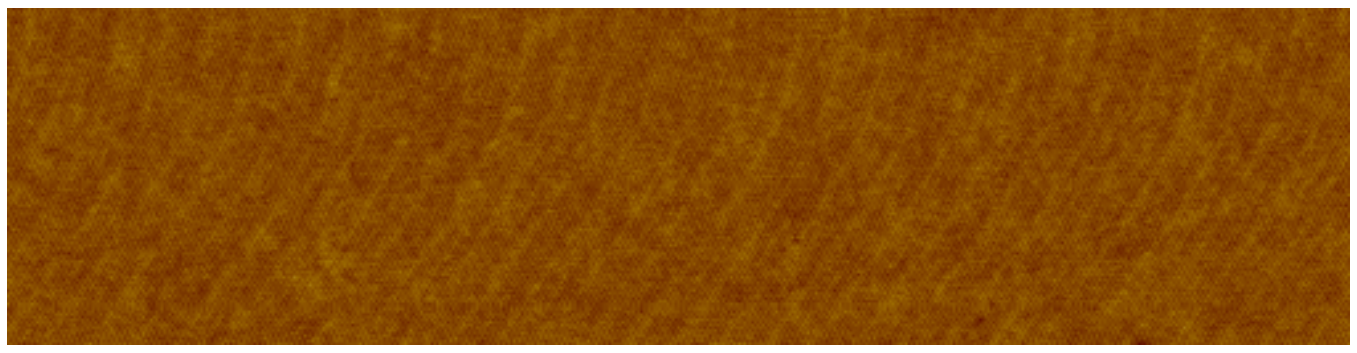 | 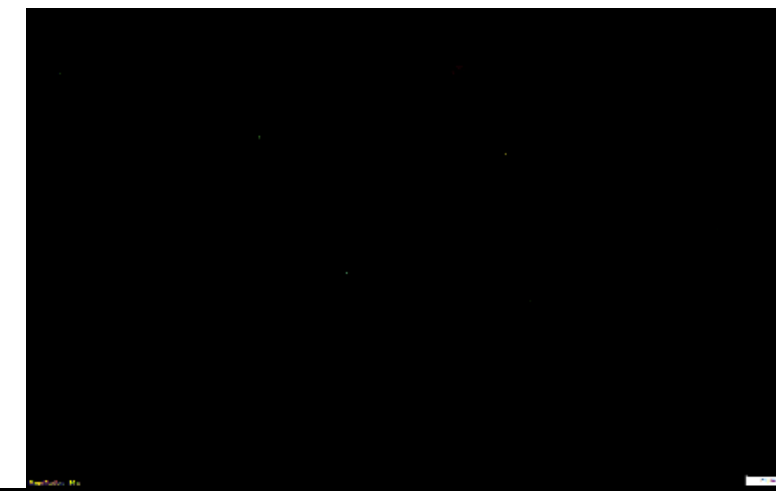 |
| 1400 | 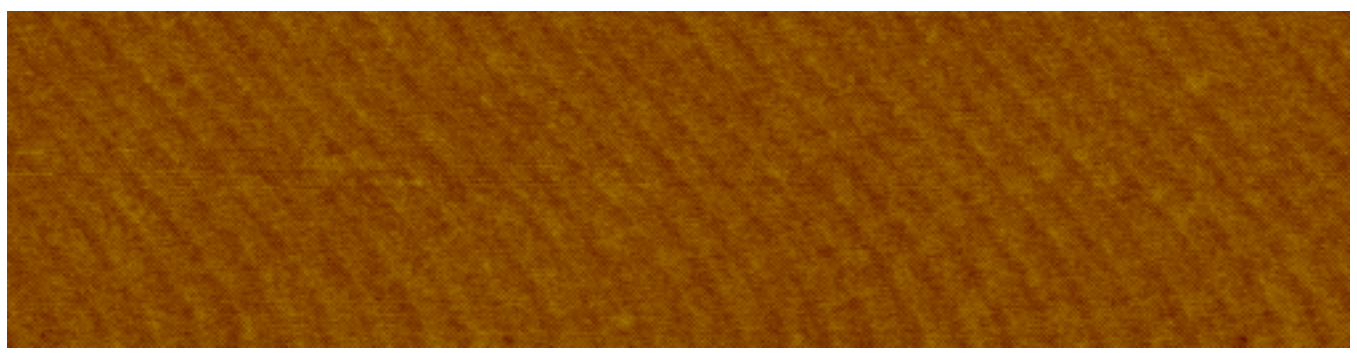 | 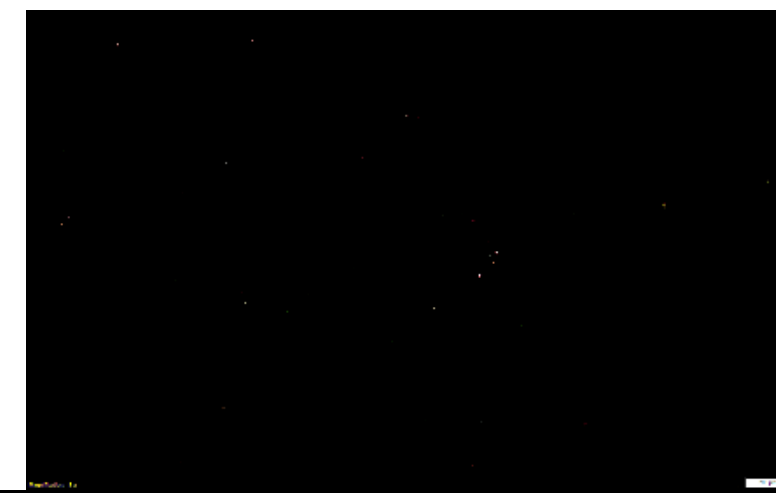 |
| 2200 | 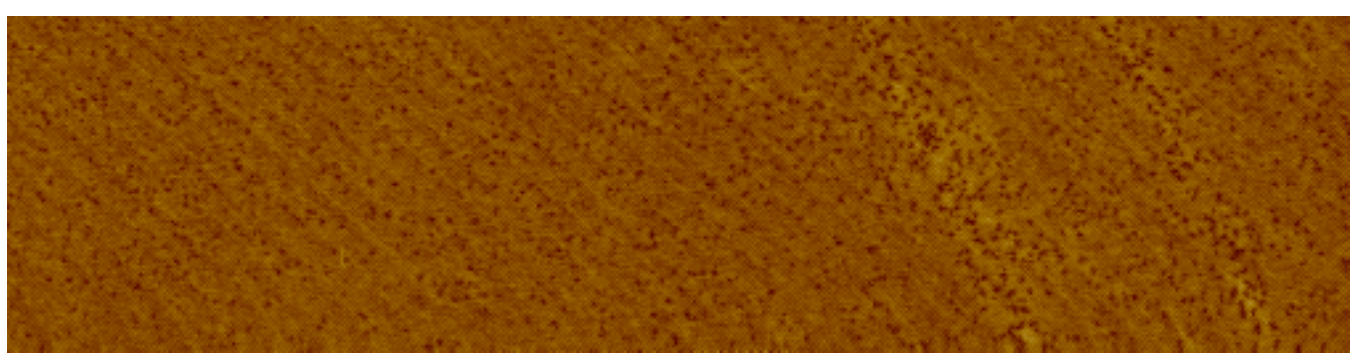 | 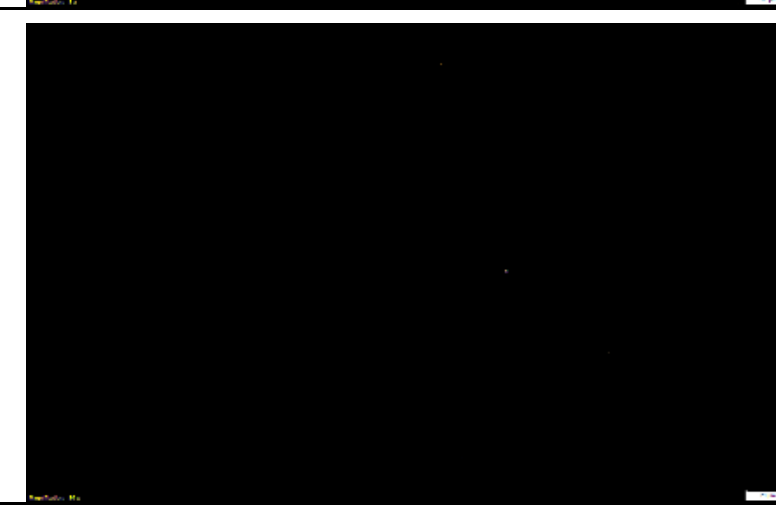 |
| 3500 | 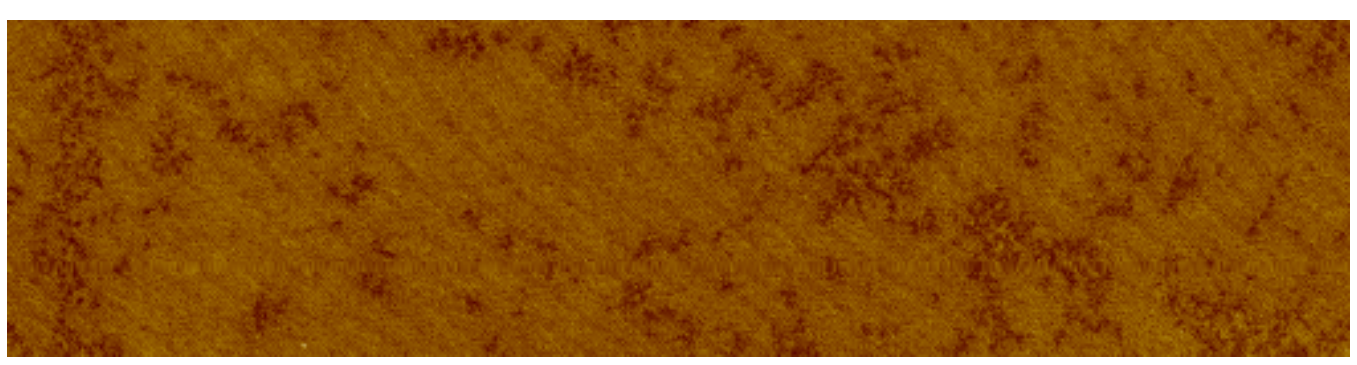 | 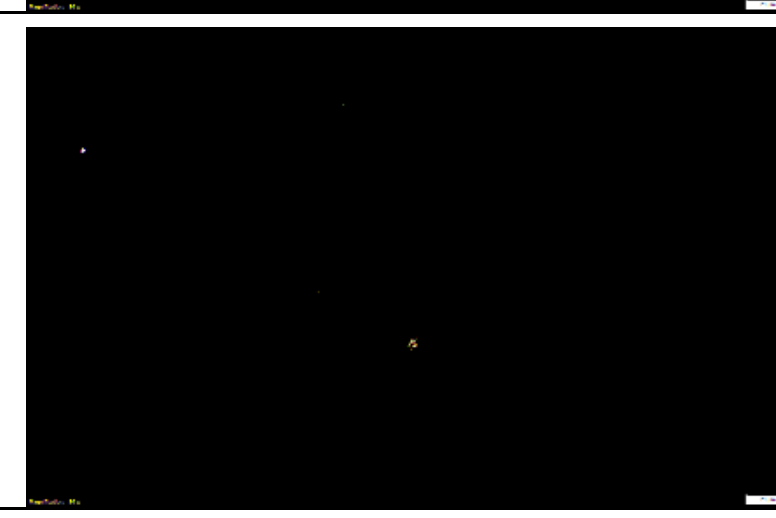 |
| 4800 | 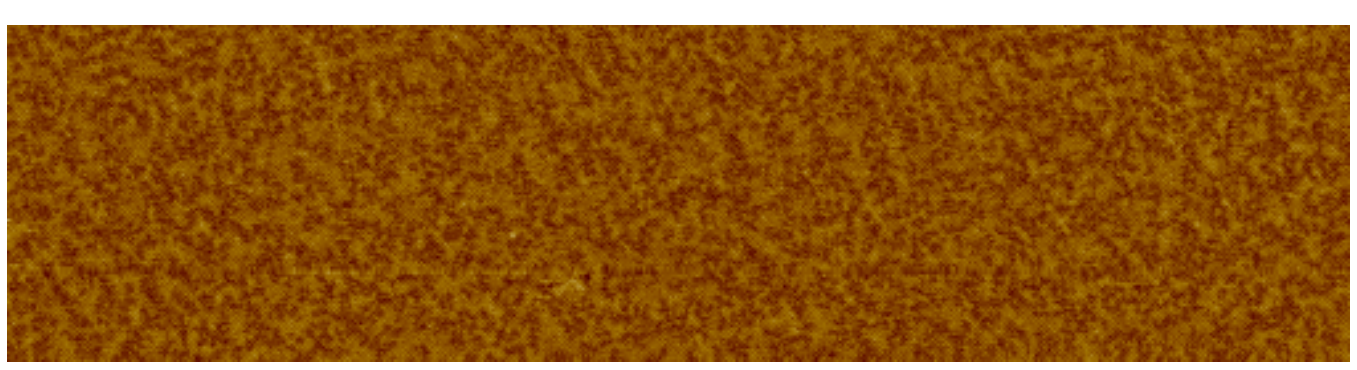 | 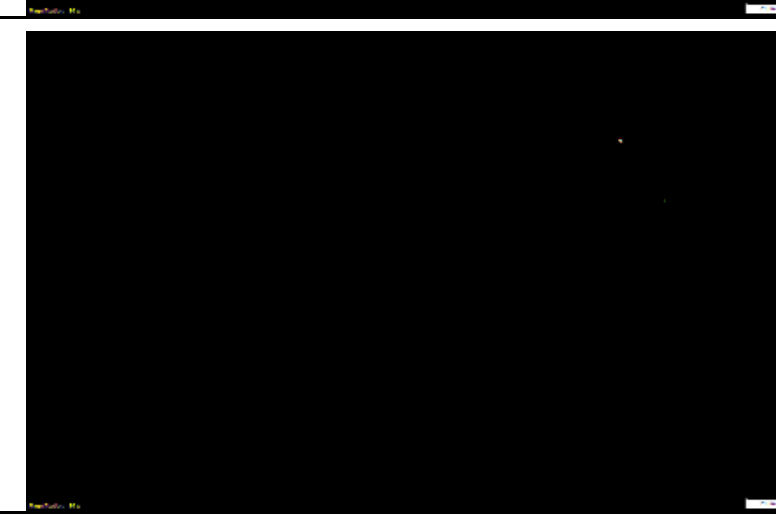 |
| 6000 | 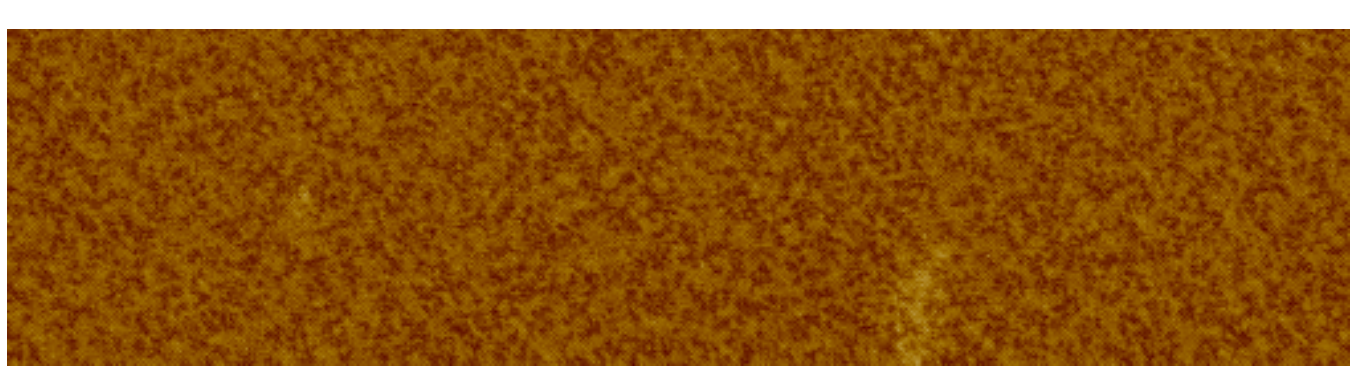 | 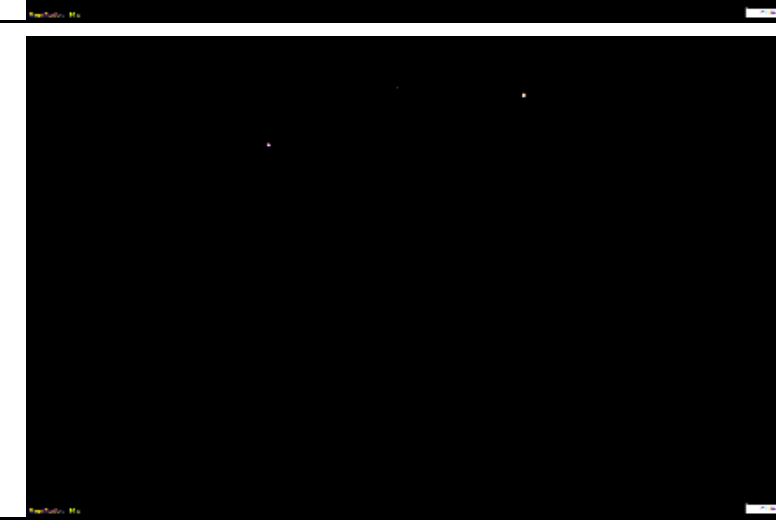 |

**Table 8S.** AFM Images (size: 5 x 1.25 µm; data scale: 10 nm) and optical images (size: 362 µm x 226 µm) of spin-coated 0.2 wt% PVP on C8 (15 min).

| spin rate | AFM | optical |
|---|---|---|
| 900 | 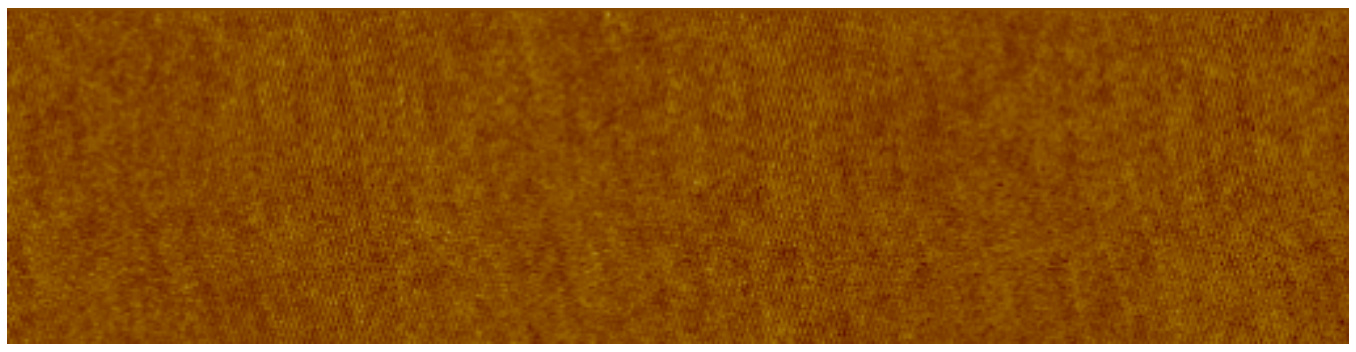 | 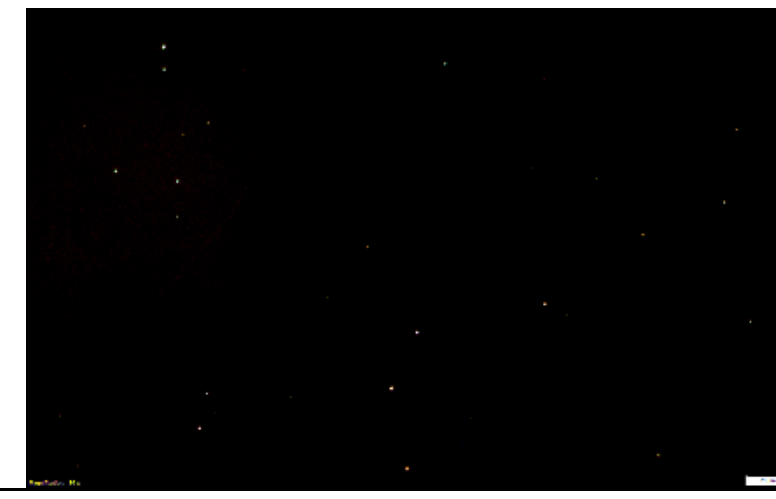 |
| 1400 | 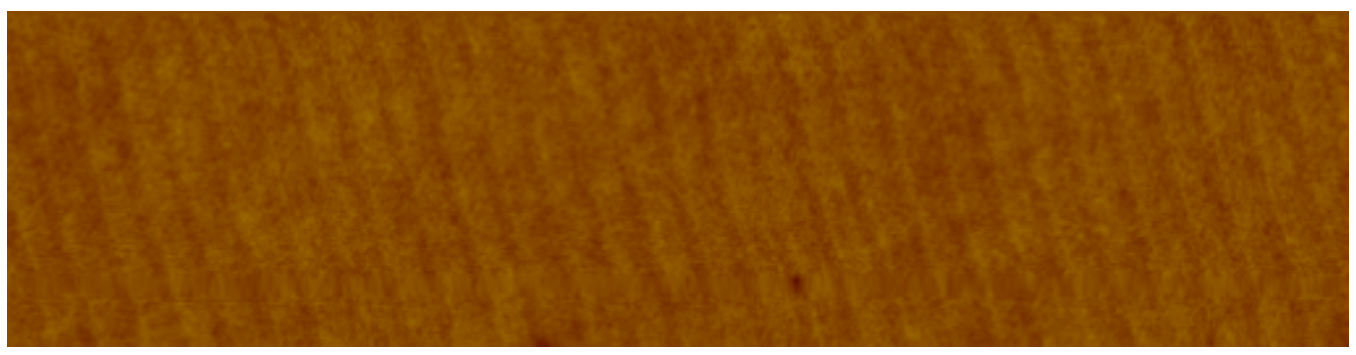 | 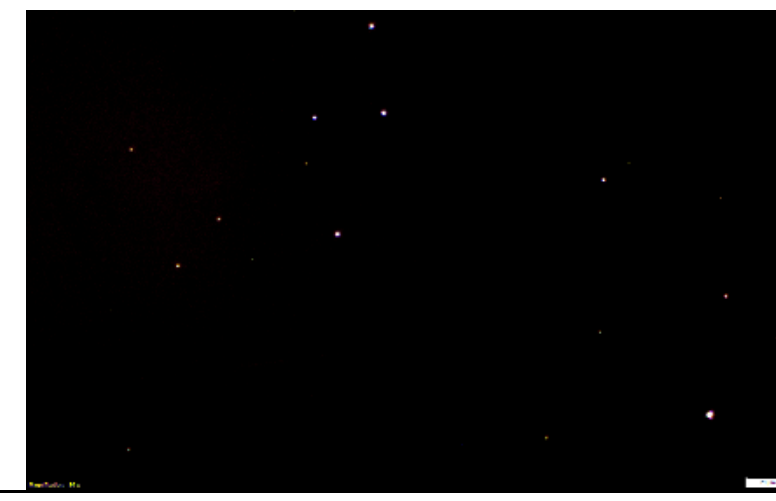 |
| 2200 | 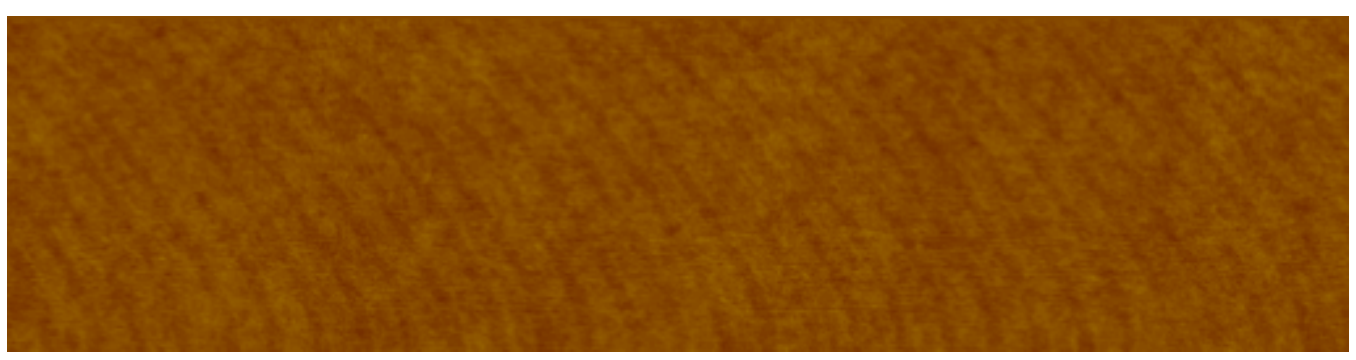 | 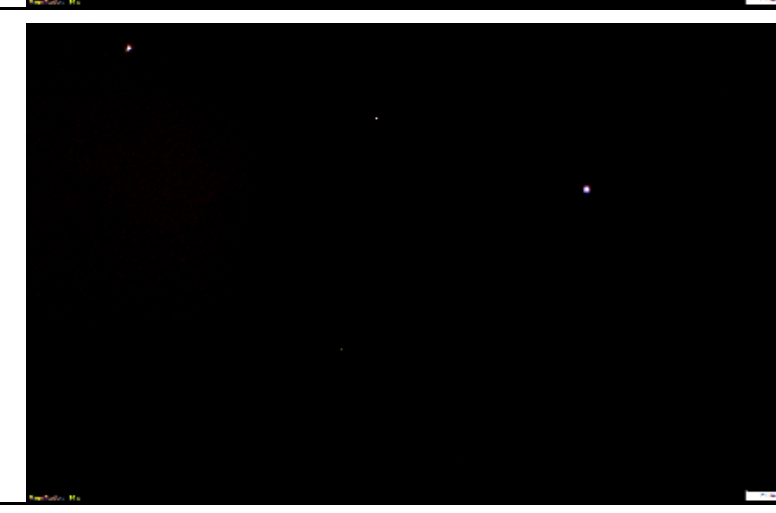 |
| 3500 | 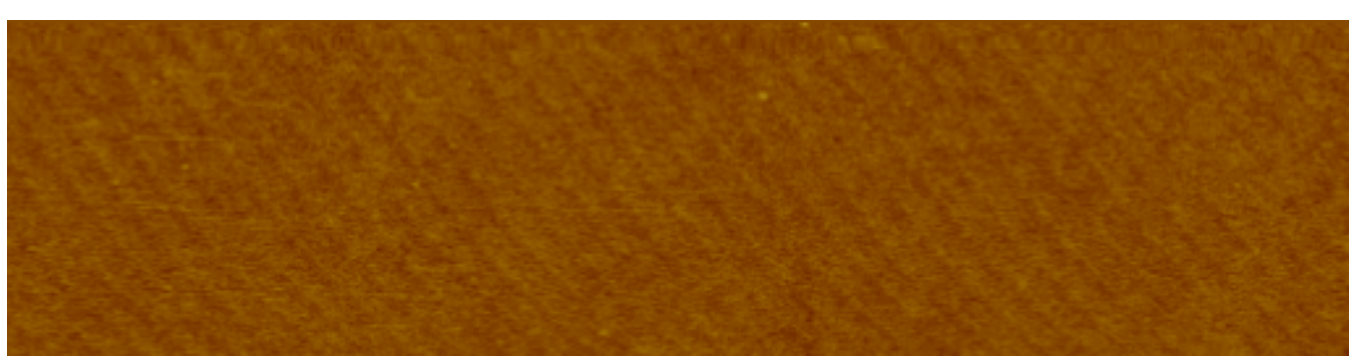 | 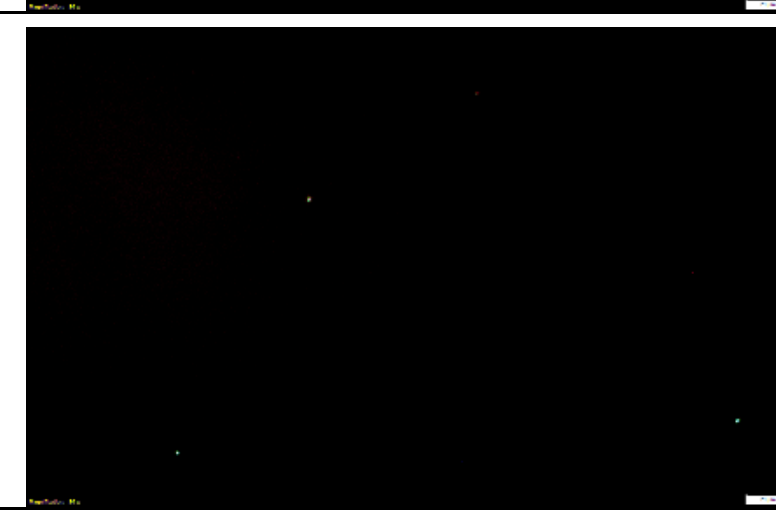 |
| 4800 | 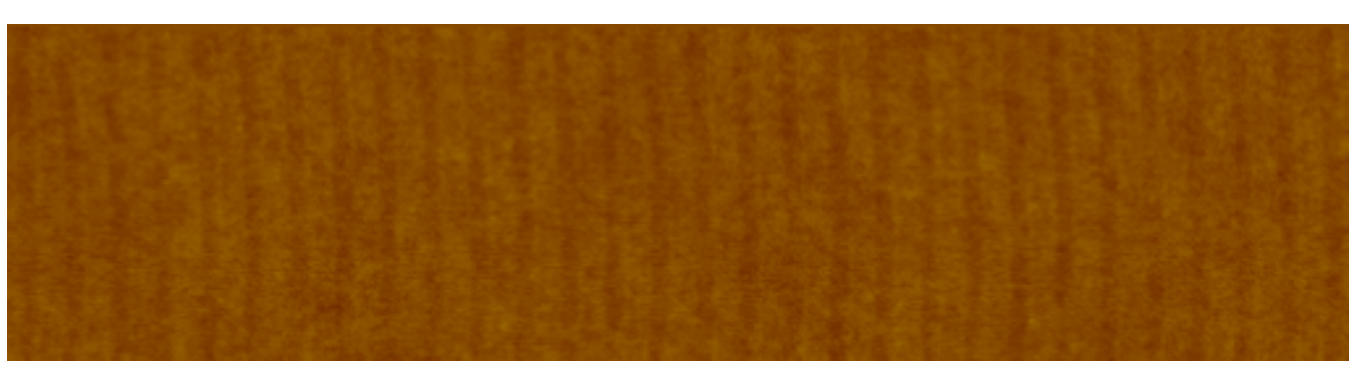 | 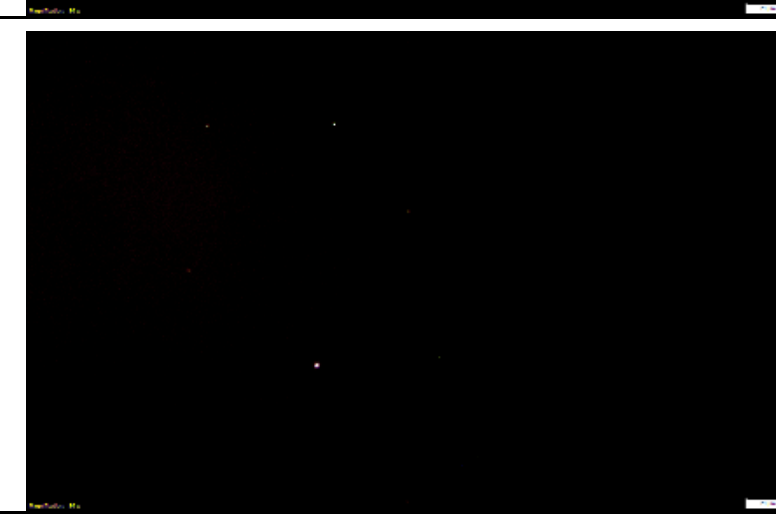 |
| 6000 | 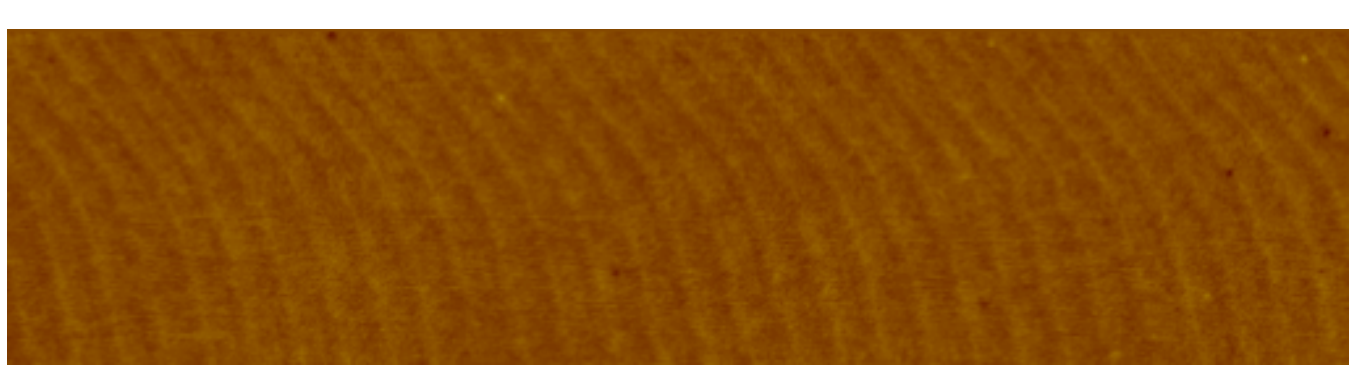 | 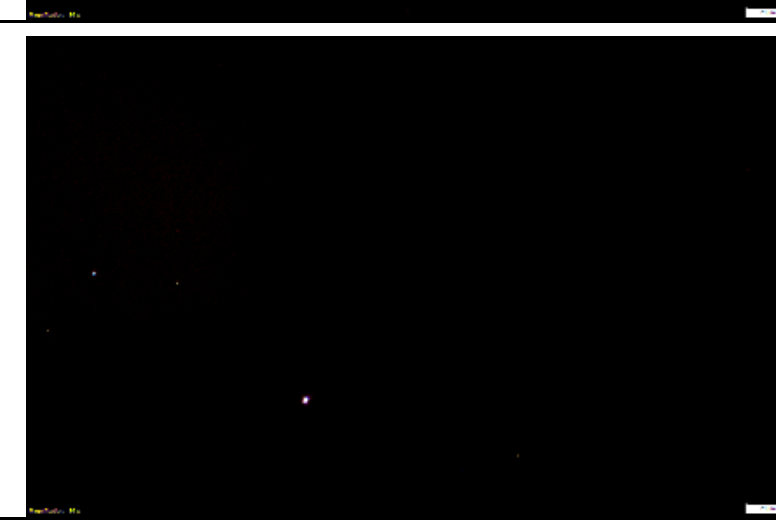 |
